# The newly developed porcine-origin parainfluenza virus PIV5-JS17 serves as an exogenous gene delivery system for swine

**DOI:** 10.1128/jvi.01858-25

**Published:** 2026-01-13

**Authors:** Guangyi Cong, Huan Li, Liang Li, Jianfei Chen, Fang Fu, Huiwei Deng, Zedong Hu, Linan Wang, Yijing Li, Mei Xue, Li Feng

**Affiliations:** 1 State Key Laboratory of Animal Disease Control and Prevention, Harbin Veterinary Research Institute, Chinese Academy of Agricultural Sciences 687216 , Harbin, Heilongjiang, China; 2 College of Veterinary Medicine, Northeast Agricultural University 12430, Harbin, Heilongjiang, China; Emory University School of Medicine, Atlanta, Georgia, USA

**Keywords:** parainfluenza virus type 5, gene delivery, viral vector, exogenous gene expression cassette, optimal insertion site

## Abstract

Emerging and re-emerging swine viral infectious diseases impose substantial economic burdens. Additionally, swine, which frequently interact with humans, may facilitate virus evolution, posing a risk to public health security. Consequently, there is a pressing need to develop safe, effective, and rapid vaccine platforms, with vector vaccine being a viable option. In this study, we utilized the enhanced green fluorescent protein (eGFP) as an exogenous reporter to investigate the intergenic regions of the PIV5-JS17 strain for expressing exogenous proteins. These regions included N-P, P-M, M-F, F-SH, SH-HN, and HN-L. Our findings revealed that exogenous gene expression varied at different positions, with the expression cassette containing the non-coding sequence within the P-M intergenic region achieving the highest eGFP fluorescence intensity. Then, we successfully established a porcine infection model through oral administration of the recombinant virus, identified the target organs infected, and verified the safety of the viral vector in swine. Furthermore, using porcine deltacoronavirus (PDCoV)-S protein as a model antigen, it was demonstrated that the recombinant virus triggered a robust humoral and cellular immune responses. In conclusion, we have developed a novel oral gene delivery system for swine, providing insights and guidance for the design of vector vaccines based on the newly discovered porcine PIV5, the selection of appropriate exogenous gene insertion sites, and vaccine delivery strategies.

**IMPORTANCE:**

The research presented in this paper hinges on the fortunate isolation of the PIV5-JS17 strain from the intestines of pigs. Given the pressing demand for oral vaccines, the emergence of this novel PIV5 strain capable of infecting both the respiratory and intestinal tracts has sparked our interest in developing it as a vector vaccine. Utilizing eGFP as a model exogenous gene, our findings reveal that the P/M intergenic region serves as the optimal site for the insertion of exogenous genes. Using the PDCoV-S protein as a model antigen, the study shows that this novel porcine-derived PIV5 virus vector presents innovative prevention methods and gene delivery strategies for addressing porcine infectious diseases.

## INTRODUCTION

Numerous viruses infect the intestinal and respiratory systems of pigs, causing significant losses to the swine industry. Some of these viruses are zoonotic, posing a serious threat to human health. Vaccination is an effective method for preventing and controlling viral infections. Vaccines can be classified into several categories, including attenuated live vaccines, inactivated vaccines, subunit vaccines, vector vaccines, nucleic acid vaccines, and nanoparticle vaccines. Traditional immunization against swine diseases has relied on inactivated or attenuated vaccines administered via intramuscular injection, which is both time-consuming and labor-intensive. Furthermore, the use of syringes can introduce secondary contamination. Consequently, the development of oral immunization holds the potential to revolutionize the swine industry by providing a more efficient and safer alternative to traditional vaccination methods. In the absence of adjuvants, vector vaccines, due to their inherent infectivity, can be administered orally or via nasal spray, enabling them to infect through the mucosa and achieve the vaccination objective while stimulating the production of secretory IgA (SIgA) in the mucosa (1).

Over the past few decades, viral vector vaccines have undergone substantial advancements. These viral vectors include poxviruses, adenoviruses, vesicular stomatitis virus, Newcastle disease virus (NDV), rabies virus, measles virus, influenza virus, and parainfluenza virus (2–9). They are either non-pathogenic or exhibit only mild pathogenicity, often manifesting as transient infections. For Severe Acute Respiratory Syndrome Coronavirus 2 (SARS-CoV-2), memory CD8+ T cells may provide more lasting protection than memory plasma cells. This suggests that viral vector vaccines, in addition to eliciting humoral immunity akin to inactivated vaccines, also mediate cellular immunity by virtue of their ability to infect hosts (10).

Parainfluenza virus type 5 (PIV5) belongs to the *Rubulavirus* genus within the Paramyxoviridae family (11). It is a single-stranded, negative-stranded RNA virus with a 15,246 bp genome, adhering to the “rule of six” (the genome length of viral nucleic acids is divisible by six) (12). Its mRNA transcribes from the 3′ to 5′ end, flanked by a 3′-leader and a 5′-tailer (11). The classic PIV5-W3A strain encodes eight viral proteins: nucleocapsid protein (NP), phosphoprotein (P), V protein (V), matrix protein (M), fusion protein (F), small hydrophobic protein (SH), hemagglutinin‐neuraminidase (HN), and polymerase large protein (L) (12). The P and V proteins share an ORF, with two non-templated G insertions during transcription due to ORF sliding. This results in two proteins with shared C-terminals but distinct N-terminals (13). However, some porcine PIV5 strains, such as KNU-11, lack the SH protein, possessing only seven virulence proteins (14). Viral genomes contain non-coding regions between coding regions, including the gene end (GE), intergenic regions (IG), and gene start (GS) signals, which affect gene expression in coding regions by regulating transcription and translation (15, 16).

PIV5 infects a wide range of species, including rats, ferrets, guinea pigs, monkeys, humans, pigs, dogs, and lesser pandas (12, 14, 15, 17–22). It is also biologically safe enough to be studied in most laboratories worldwide without the need for high-level biosafety laboratories. PIV5 adapts well to various cell types *in vitro* and has potential as a viral vector (23). The diameter of PIV5 ranges from 80 to 200 nm and appears pleomorphic under an electron microscope, which is conducive to the precise expression of the topological structure of exogenous proteins (12). As a commercially promising viral vector, PIV5 is notable for its lack of a DNA stage in its life cycle, preventing genetic modification of host cell DNA resulting from recombination or insertion (12).

When PIV5 serves as a viral vector, such as other paramyxoviruses, exogenous genes should be inserted into its non-coding regions. To activate their expression, GS signal and GE signal are added to the 5′ end and 3′ end, respectively, adhering to the “rule of six” to form an expression cassette that triggers the correct biological activity (17, 24, 25). Due to the polarity mechanism of transcription in single-stranded negative-strand RNA viruses, viral gene expression decreases from the 3′ to 5′ end. However, studies show that exogenous genes inserted into these viruses do not always follow this rule; for instance, optimal insertion sites for NDV and avian paramyxovirus type 3 lie between the P and M gene (5, 26). The biological activities of various paramyxoviruses differ, and the optimal exogenous protein insertion site for porcine PIV5 virus, as well as its mechanism, remains unclear. Additionally, no vaccine based on the porcine PIV5 vector has been developed yet.

In this study, we used a reverse genetics system to insert eGFP at various positions of PIV5-JS17, finding the P/M intergenic region as the best insertion site. Using recombinant viruses, we established a porcine infection model via oral route, confirming no severe diseases in pigs. Subsequently, we constructed a recombinant virus expressing the PDCoV-S protein at this site. Importantly, the vaccine induced robust antigen-specific humoral and cellular immune responses. Thus, the data presented here indicate that the PIV5-JS17-based vector is a promising and immunogenic vaccine candidate for swine.

## RESULTS

### Isolation and identification of the novel porcine PIV5 strain JS17

**Fig 1:**
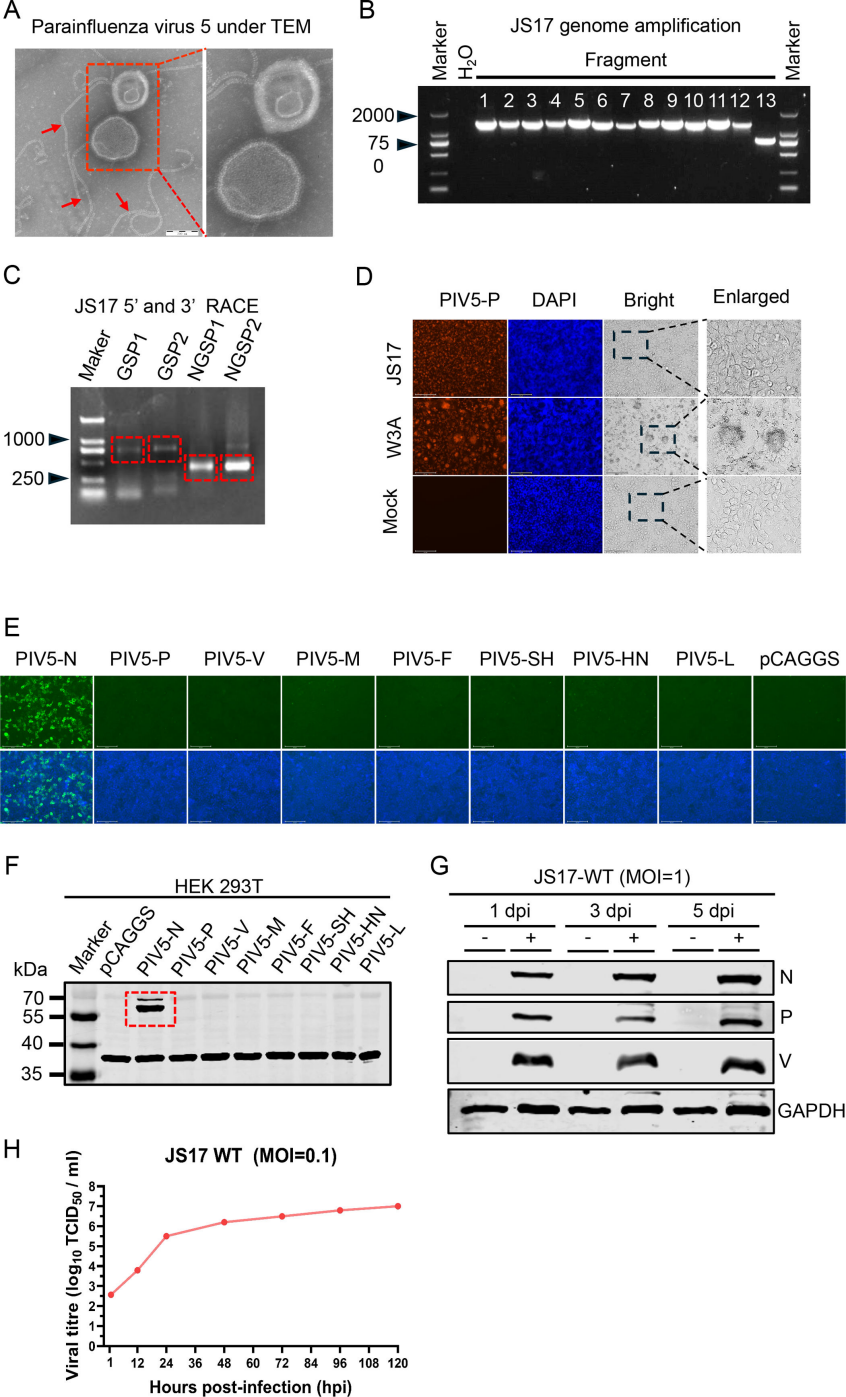
Isolation and identification of the JS17 strain of PIV5. (**A**) The virus particles were observed through an electron microscope. (**B**) PCR amplification of the entire PIV5 genome was divided into 13 fragments. (**C**) Rapid amplification of cDNA ends (RACE) for the 5′ and 3′ ends of the JS17 strain of PIV5 was performed using gene-specific primers (GSP1/NGSP1 for 5′ RACE and GSP2/NGSP2 for 3′ RACE). (**D**) Vero E6 cells were infected with either the JS17 strain or W3A strain of PIV5 (MOI = 0.1), or mock-infected. The replication of PIV5 was measured by IFA using mAb against PIV5-P protein (red). (**E**) Production of a mAb against the JS17 strain of PIV5 N protein (named 10B12). (**F**) Confirmation of the mAb by Western blotting. (**G**) Western blotting analysis of the JS17 infection efficiency in Vero E6 cells (MOI = 1) at 1, 3, and 5 dpi. (**H**) Growth kinetics of the JS17 in Vero E6 cells infected at an MOI of 0.1. Virus titers in the media collected daily for 5 days were determined by IFA.

Fecal samples collected from piglets with diarrhea were tested, revealing no presence of common piglet diarrhea viruses, such as porcine epidemic diarrhea virus (PEDV), transmissible gastroenteritis virus (TGEV), PDCoV, or porcine rotavirus (PoRV) (data not shown). The fecal supernatant was inoculated into Vero E6 cells containing 5% penicillin, gentamicin, and streptomycin. After five consecutive passages, the fifth-generation cell cultures were subjected to supercentrifugation and observed under a transmission electron microscope (TEM), revealing paramyxoviriform particles with a diameter ranging from 80 to 200 nm (Fig. 1A). Moreover, typical viral nucleocapsids were also observed (indicated by red arrow) (Fig. 1A). Based on the TEM results, we suspected that the unknown virus might be PIV5 and designed whole-genome primers to sequence the entire genome, including the 5′ and 3′ ends (Fig. 1B and C). To precisely determine the specific nucleotides at the termini of the PIV5 sequence, we employed 5′ and 3′ rapid amplification of cDNA ends (RACE) technology. Following the manufacturer’s protocol, gene-specific primer 1 (GSP1) was used for 5′-RACE and gene-specific primer 2 (GSP2) for 3′-RACE, with both serving as the outer primers. Corresponding nested gene-specific primers (NGSP-1 and NGSP-2) were used as the inner primers for GSP1 and GSP2, respectively. The target bands are indicated within the red boxes (Fig. 1C). We assembled the measured sequences. The sequence has been deposited in GenBank (accession number OR888917.1). Compared with the W3A strain, the JS17 strain did not induce syncytia *in vitro* (Fig. 1D).

To identify the isolation results of the JS17 strain, indirect immunofluorescence with a monoclonal antibody (mAb) against the P protein showed specific fluorescence in PIV5-JS17-infected cells (Fig. 1D). Moreover, a mAb against the N protein was prepared (Fig. 1E and F). The expression levels of N, P, and V proteins in PIV5-infected cells increased in a time-dependent manner, as confirmed by Western blotting (Fig. 1G). Further, to assess the growth kinetics of this novel PIV5 strain, it was inoculated into Vero E6 cells at a multiplicity of infection (MOI) of 0.1. A one-step growth curve analysis showed that the production of infectious virions progressively increased, peaking at 10^7^ TCID_50_/mL on the fifth day. Together, our results confirmed the isolation of PIV5-JS17 from the fecal samples of piglets.

### Whole-genome sequence analysis and phylogenetic analysis

**Fig 2:**
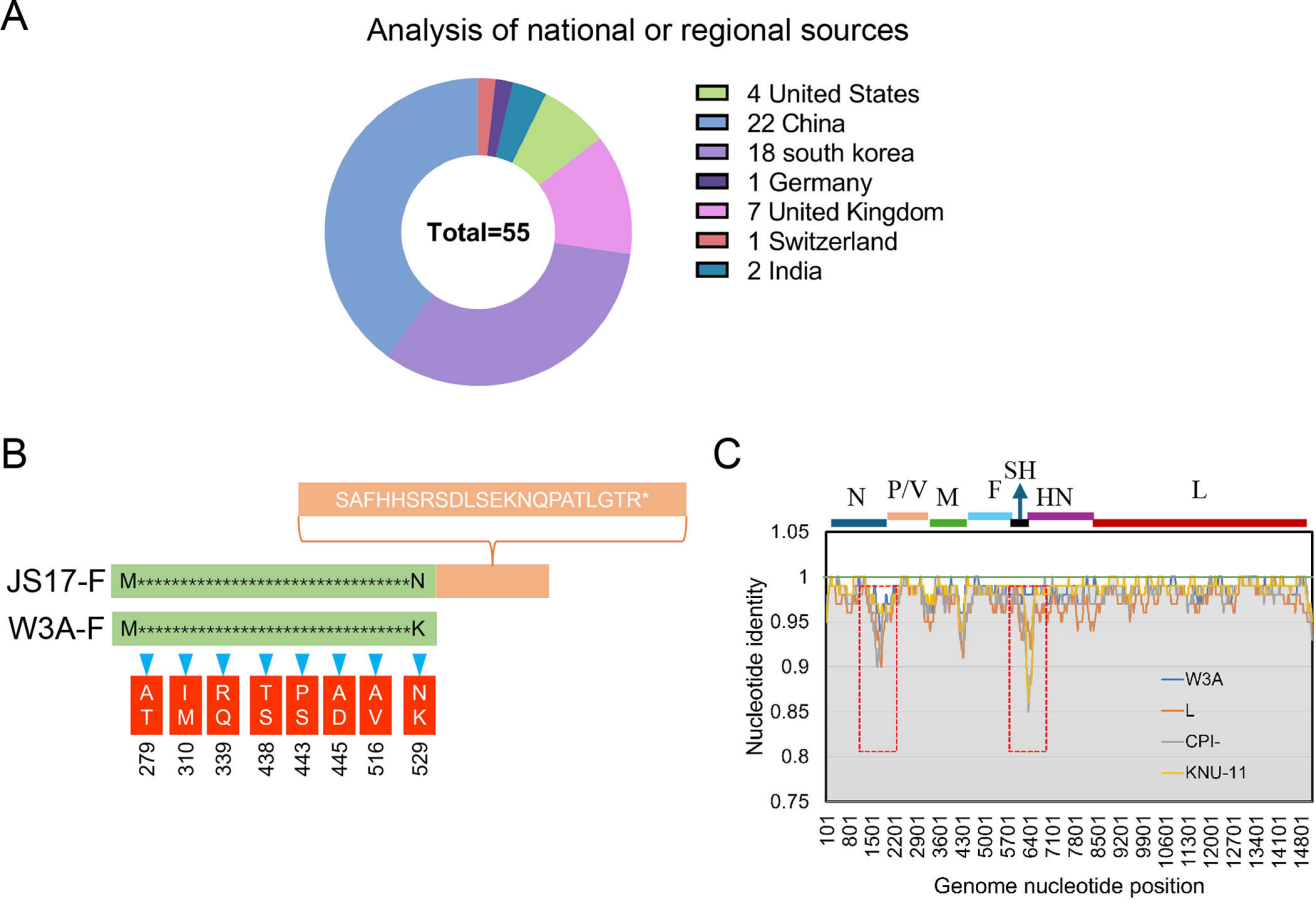
Genomic characterization of JS17. (**A**) The countries/regions of isolation for the PIV5 strain are represented by distinct colors. (**B**) Compared to the classic W3A strain, JS17 has the highest number of amino acid mutations in its F protein. Notably, its C-terminal region (highlighted in orange) has an additional 22 amino acids. (**C**) Nucleotide similarity profiles are shown for representative strains from different species.

We analyzed all PIV5 whole genomes deposited in GenBank prior to January 1, 2024. A total of 55 sequences were available, excluding two sequences of the W3A strain (only one of the three was used) and the Moskva strain from Russia (due to significant sequence gaps). These sequences originated from various countries and regions, including the United States (four strains), China (22 strains), South Korea (18 strains), the United Kingdom (seven strains), Germany (one strain), Switzerland (one strain), and India (two strains) (Fig. 2A).

To gain deeper insights into the genetic characteristics of the PIV5-JS17 strain, we sequenced its entire genome, which is 15,246 nucleotides long, aligning with the majority of PIV5 strains globally. Notably, only the LN (Homo sapiens, GenBank: JQ743324.1) and RQ (Homo sapiens, GenBank: JQ743327.1) strains, isolated in the UK in 1976 and 1980, respectively, have a slightly longer genome of 15,252 nucleotides (15). The PIV5-JS17 genome comprises a 3′ leader sequence, followed by the NP, P/V, M, F, SH, HN, and L genes, and terminates with a 5′ tailer sequence, totaling seven genes.

To ascertain the amino acid and nucleotide similarity of the JS17 strain compared to other PIV5 strains, we conducted an analysis involving 55 PIV5 strains. Notably, the 13 PIV5 strains with the highest nucleotide similarity to JS17, ranging from 99.97% to 99.78% (Table S1), all originated from China, suggesting a potential national or regional distribution pattern of PIV5. The second-most similar strain was found in ticks (99.91%), whereas the three most divergent strains were from pigs (96.89%–96.71%), hinting that nucleotide similarity may not be species-dependent. The nucleotide and amino acid identities of various PIV5 genes are as follows: N (96.71%–100%/98.0%–100%), P (93.9%–100%/96.77%–100%), V (96.4%–100%/96.86%–100%), M (96.0%–100%/96.0%–100%), F (95.1%–100%/95.89%–100%), SH (84.4%–100%/absent in some strains), HN (97.3%–99.8%/97.53%–99.82%), and L (97.84%–99.97%/98.4%–100%). Notably, the absence of the SH gene in some strains suggests that it may not be essential for the PIV5 life cycle (Table S1). Compared to the classical W3A strain, the most significant amino acid difference is in the F protein, where a stop codon changes from “TAA” to “TCA” results in an additional 22 amino acids in the JS17 strain (Fig. 2B). To analyze genes with notable differences among PIV5 strains from different species, we selected four representative strains (16, 21, 31, and 32). The largest differences are in sequences encoding the C-terminus of N and the P/V junction (Fig. 2C).

The phylogenetic analysis of the whole genome categorized PIV5 strains into Group A and Group B (Fig. S1). Most porcine PIV5 strains belonged to Group A, with only a few in Group B. All human PIV5 strains clustered in Group A, indicating homologous genes or a similar origin despite originating from different countries. The PIV5-JS17 strain is evolutionarily close to two Group A strains (OK505006.1 and MW051776.1), suggesting a shared “ancestor” strain. This implies that tick PIV5 (MW051776.1) might result from ticks feeding on PIV5-infected pigs. Notably, the PIV5-JS17 strain was also closely related to the tiger, pangolin, and red panda strains in China. This indicates that these animals were exposed to feed or the environment contaminated with porcine PIV5, which was further confirmed by phylogenetic analysis of the F (Fig. S2A) and HN (Fig. S2B) genes.

### Generation of rPIV5-JS17 with eGFP inserted at different sites

**Fig 3:**
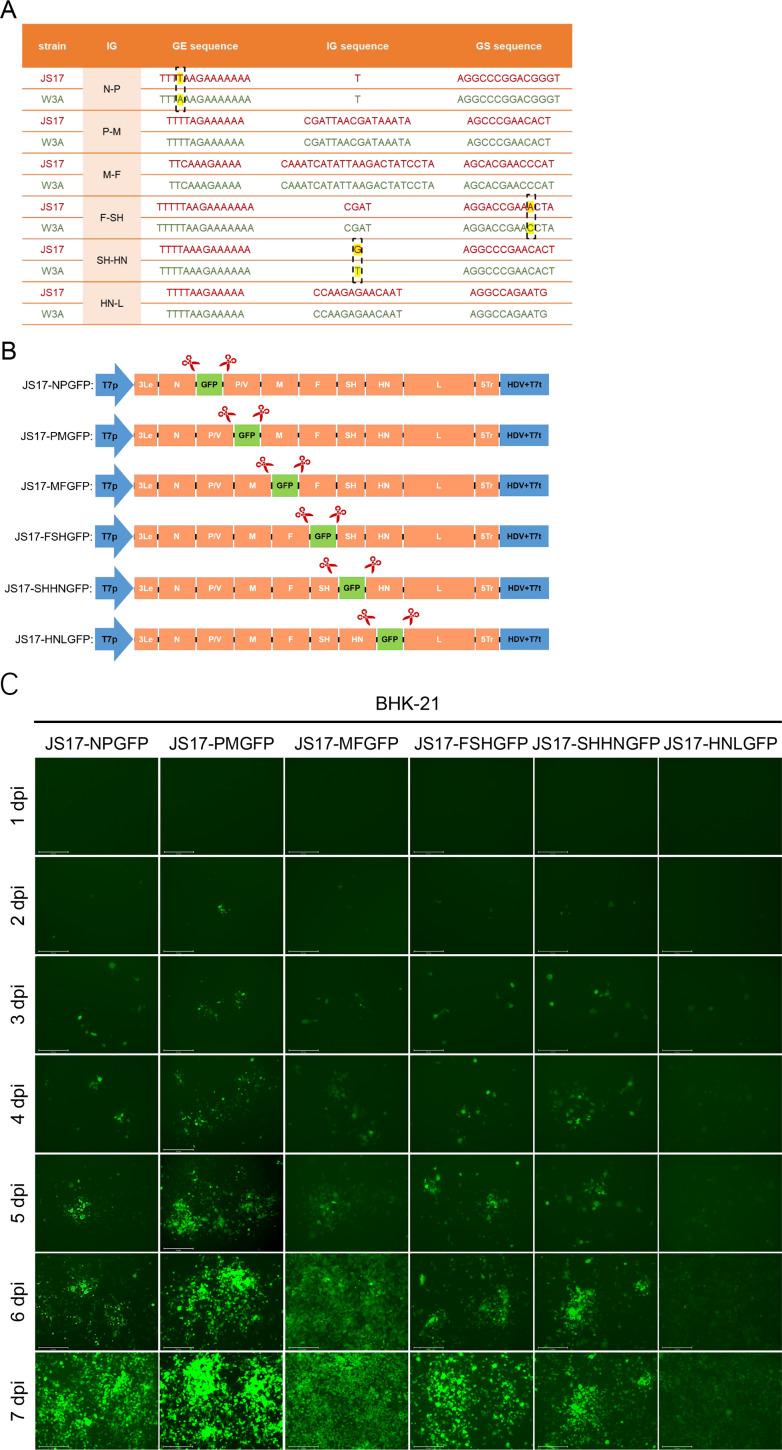
Generation of recombinant PIV5-JS17 (rPIV5-JS17) expressing eGFP. (**A**) Comparison of the intergenic regions between PIV5-JS17 and PIV5-W3A, highlighting gene ends, intergenic regions, and gene initiation. (**B**) The exogenous protein eGFP was inserted into various intergenic regions of the recombinant virus rPIV5-JS17, including N/P (JS17-NPGFP), P/M (JS17-PMGFP), M/F (JS17-MFGFP), F/SH (JS17-FSHGFP), SH/HN (JS17-SHHNGFP), and HN/L (JS17-HNLGFP). (**C**) Recombinant rPIV5-JS17 viruses with eGFP inserted at different positions (JS17-NPGFP, JS17-PMGFP, JS17-MFGFP, JS17-FSHGFP, JS17-SHHNGFP, and JS17-HNLGFP) were monitored for fluorescence expression intensity over 7 days.

To explore the best site for inserting exogenous genes into the novel PIV5-JS17 strain isolated from porcine intestines, we first analyzed the GE, IG, and GS sequences of each gene interval region (Fig. 3A). We then connected the eGFP to the GS sequence to create the corresponding exogenous gene expression cassette (Fig. 3B). Subsequently, we developed a reverse genetic system for the PIV5-JS17 strain, driven by the full PIV5-JS17 strain genome cDNA under the T7 promoter (27). All six rPIV5-JS17 variants successfully produced progeny viruses expressing eGFP, with JS17-PMGFP exhibiting the brightest fluorescence at 7 dpi (Fig. 3C).

### The P/M gene intergenic region is identified as the best site for inserting foreign genes into rPIV5-JS17

**Fig 4:**
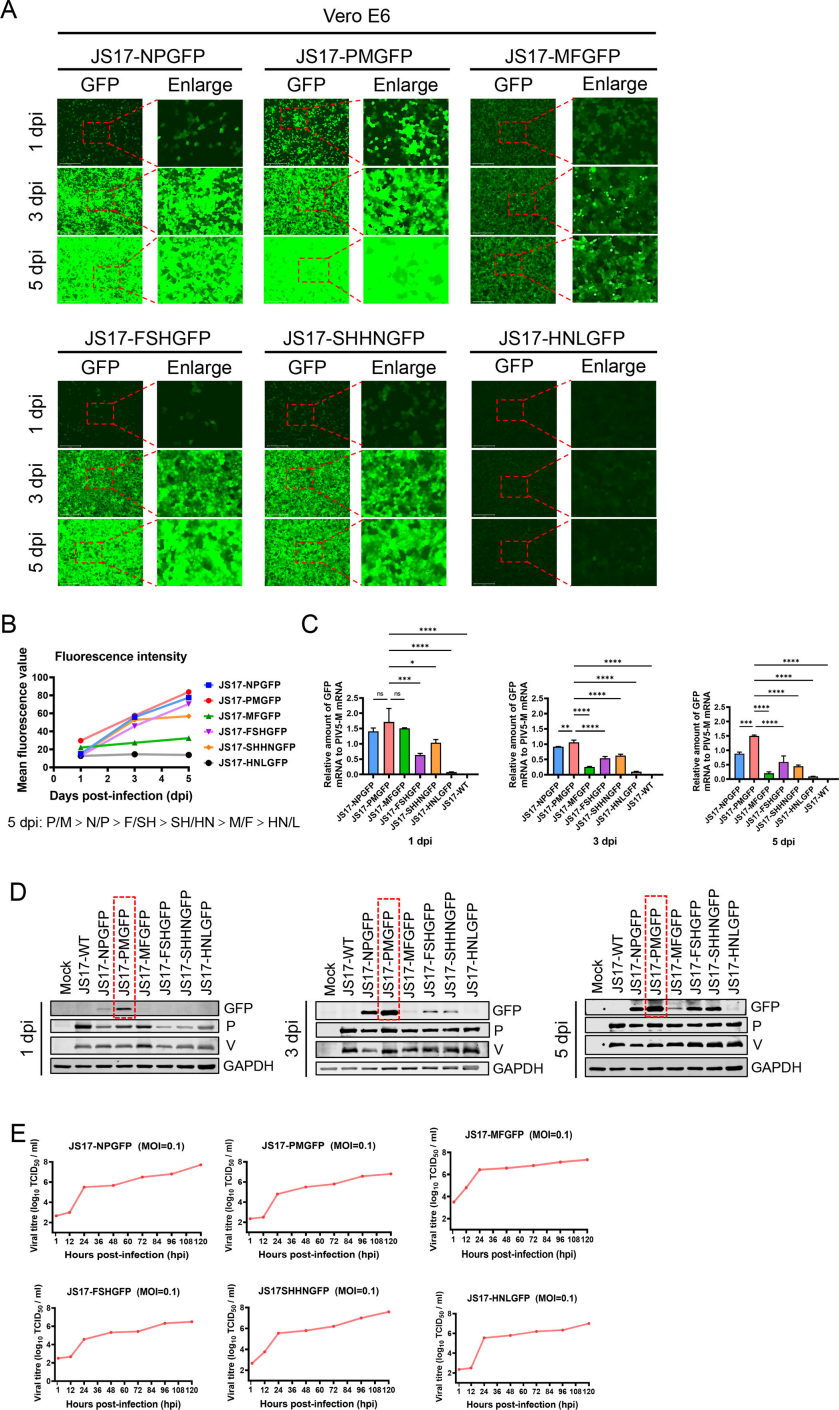
Comparison of eGFP fluorescence intensity among different rPIV5-JS17. (**A**) Vero E6 cells infected with JS17-NPGFP, JS17-PMGFP, JS17-MFGFP, JS17-FSHGFP, JS17-SHHNGFP, and JS17-HNLGFP at an MOI of 1 were monitored for fluorescence intensity at 1, 3, and 5 dpi. (**B**) Fluorescence intensities were analyzed using Evos Analysis software. (**C**) RT-qPCR was performed to quantify eGFP, JS17-M, and GAPDH gene expression. (**D**) Western blotting was used to assess the expression of eGFP, JSI7-P, and JSI7-V. (**E**) Growth kinetics of different rPIV5-JS17 variants were analyzed by infecting Vero E6 cells at an MOI of 0.1 and measuring virus titers through fluorescence every 12 h. Data are presented as means ± SD (*P < 0.05; **P < 0.01; ***P < 0.001; ****P < 0.0001; ns, not significant).

To validate the P/M gene spacer region as the best insertion site for exogenous genes, we infected Vero E6 cells (MOI = 1) with six rPIV5-JS17 variants (JS17-NPGFP, JS17-PMGFP, JS17-MFGFP, JS17-FSHGFP, JS17-SHHNGFP, and JS17-HNLGFP) expressing eGFP in different IG (N/P, P/M, M/F, F/SH, SH/HN, HN/L). At 1, 3, and 5 dpi, we observed no significant cytopathic effect (CPE), but a gradual increase in eGFP fluorescence, indicating progressive viral infection (Fig. 4A). Quantification of eGFP fluorescence intensity using Evos Analysis software revealed the following order:

- At 1 dpi: JS17-PMGFP > JS17-MFGFP > JS17-NPGFP > JS17-SHHNGFP > JS17-FSHGFP > JS17-HNLGFP
- At 3 dpi: JS17-PMGFP > JS17-NPGFP > JS17-SHHNGFP > JS17-FSHGFP > JS17-MFGFP > JS17-HNLGFP
- At 5 dpi: JS17-PMGFP > JS17-NPGFP > JS17-FSHGFP > JS17-SHHNGFP > JS17-MFGFP > JS17-HNLGFP

Among them, JS17-PMGFP exhibited the brightest fluorescence at 1, 3, and 5 dpi (Fig. 4B), indicating that the P/M gene intergenic region is most favorable for exogenous gene expression in terms of eGFP fluorescence intensity. To confirm this, we analyzed the expression level of eGFP relative to PIV5-M mRNA, rather than relative to GAPDH, in order to avoid errors caused by slight differences in infection dose (28). At 1, 3, and 5 dpi, JS17-PMGFP showed the highest ratio among all rPIV5-JS17 samples (Fig. 4C), suggesting that the fluorescence intensity is strain-dependent rather than due to varying virus doses. Consistent with this, protein analysis revealed the highest levels of eGFP, PIV5-P, and PIV5-V proteins in JS17-PMGFP (Fig. 4D). Despite different insertion sites and varying GFP fluorescence brightness, all six rPIV5-JS17 strains exhibited similar growth kinetics (Fig. 4E).

### The stability of serial passages of JS17-PMGFP

**Fig 5:**
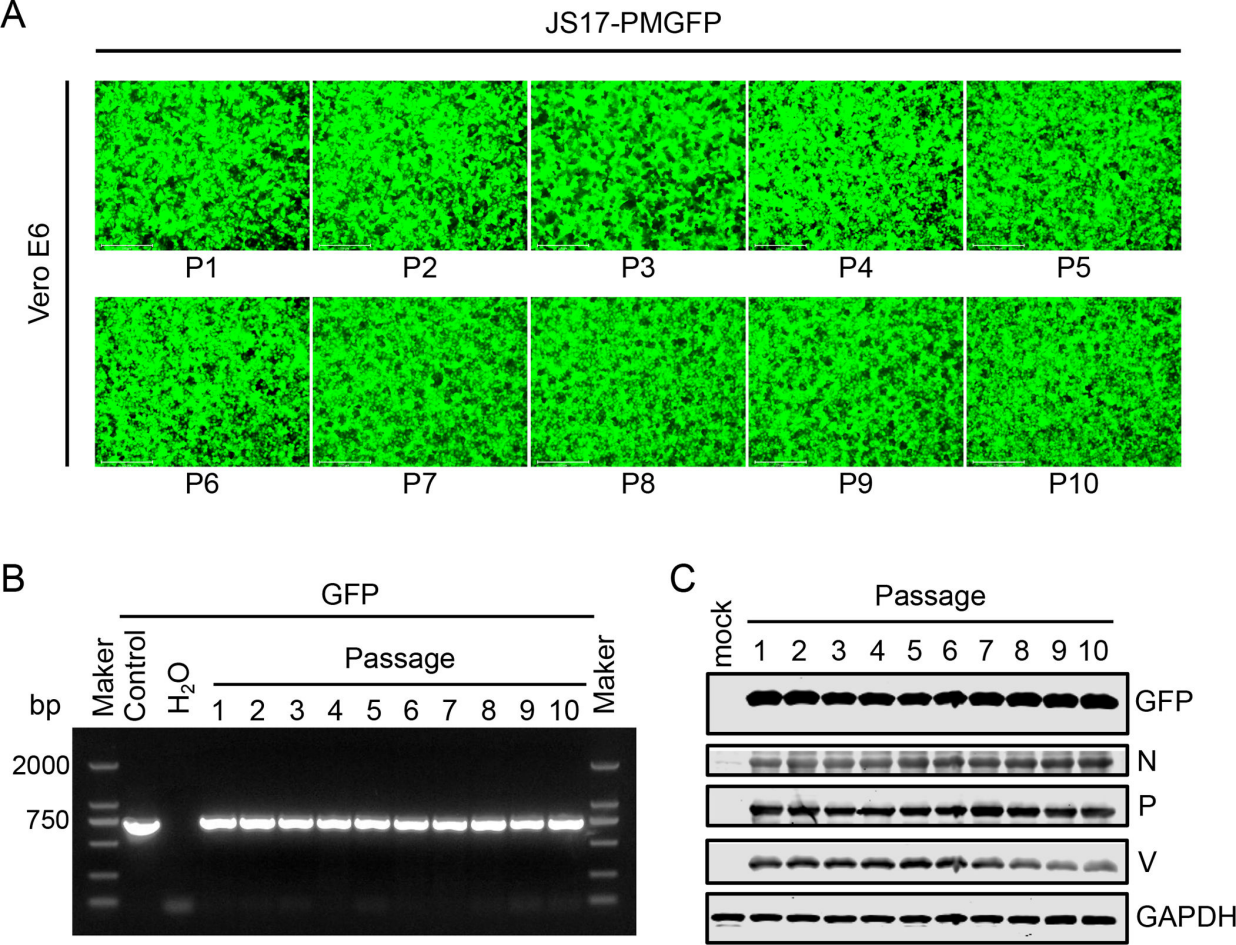
Genetic stability of JS17-PMGFP during passaging in Vero E6 cells. (**A**) The fluorescence of JS17-PMGFP (MOI = 0.1) recovered from transfected BHK-21 cells (P0) was monitored on Vero E6 cells during successive passages (P1 to P10). (**B**) Virus supernatants were collected from each passage for RT-PCR analysis. (**C**) Infected cells were harvested for Western blotting to assess protein expression.

To assess the passage stability of the eGFP protein of rPIV5-JS17, we selected JS17-PMGFP, which exhibits the brightest GFP fluorescence, and serially passaged it (MOI = 0.1) for 10 times (P1-P10). The results showed no significant change in fluorescence intensity across passages (Fig. 5A). Furthermore, RT-PCR amplification of the eGFP gene from viral RNA of JS17-PMGFP at different passages confirmed consistent gene size with expectations (Fig. 5B). Western blotting analysis of eGFP and P/V protein in various passages of JS17-PMGFP revealed that eGFP, as an exogenous protein, maintained stability in both size and expression level (Fig. 5C).

### The exogenous gene expression cassette is a key determinant of exogenous gene expression

**Fig 6:**
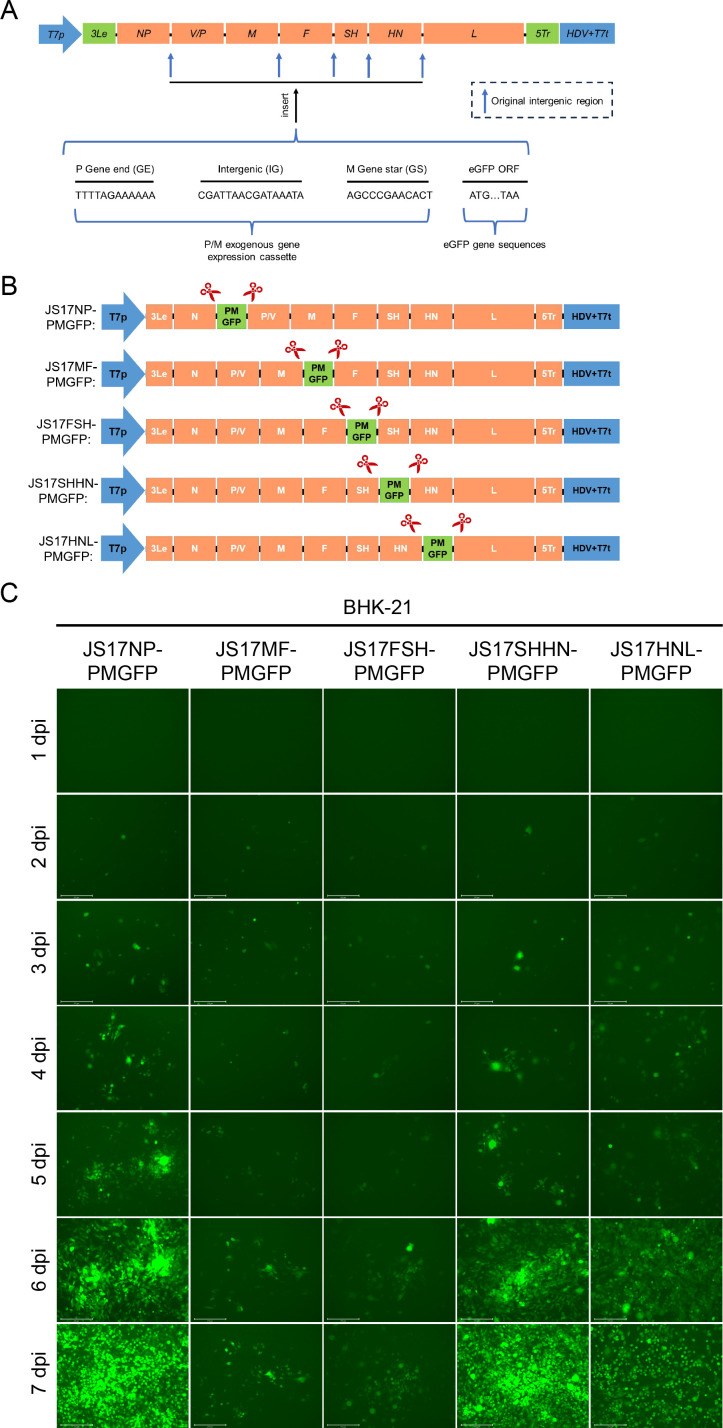
Construction of rPIV5-JS17 expressing eGFP using the PM exogenous gene expression cassette. (**A**) The eGFP gene, fused with the PM exogenous gene expression cassette, was inserted into the NP, MF, FSH, SHHN, and HNL regions of PIV5-JS17. (**B**) The resulting constructs included JS17NP-PMGFP, JS17MF-PMGFP, JS17FSH-PMGFP, JS17SHHN-PMGFP, and JS17HNL-PMGFP. The red arrow indicates the insertion site. The PM cassette comprises the P gene end, intergenic region, and M gene start. (**C**) Fluorescence expression intensity of the recombinant viruses was monitored for 7 days.

**Fig 7:**
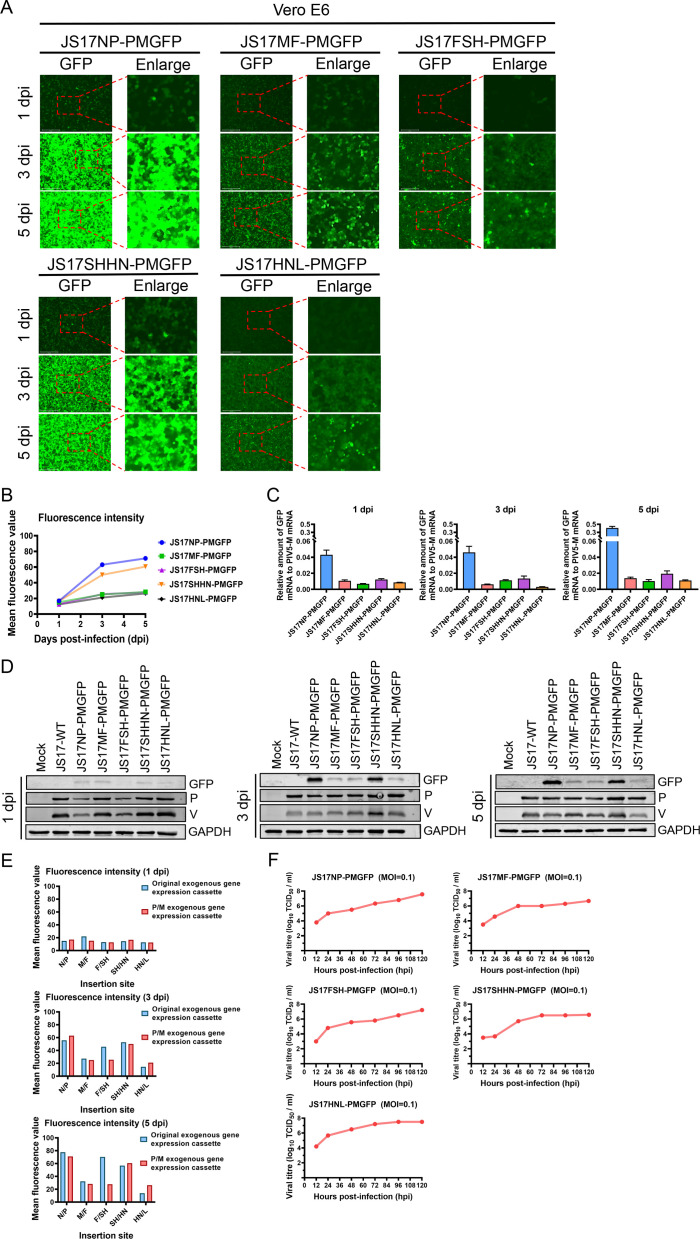
Expression of eGFP using the PM exogenous gene expression cassette in different intergenic regions of rPIV5-JS17. (**A**) Vero E6 cells were infected with recombinant viruses JS17NP-PMGFP, JS17MF-PMGFP, JS17FSH-PMGFP, JS17SHN-PMGFP, and JS17HNL-PMGFP at an MOI of 1. Fluorescence was recorded at 1, 3, and 5 dpi. (**B**) Fluorescence intensities were analyzed using Evos Analysis software. (**C**) RT-qPCR was performed to detect eGFP, JS17-M, and GAPDH gene expression. (**D**) Western blotting was used to detect protein expression at 1, 3, and 5 dpi. (**E**) Growth kinetics of the recombinant viruses were analyzed by infecting Vero E6 cells at an MOI of 0.1 and measuring virus titers through fluorescence every 12 h. (**F**) Comparison of eGFP fluorescence intensity between rPIV5-JS17 viruses using the original gene expression cassette versus the PM exogenous gene expression cassette.

To investigate the mechanism underlying the high-level expression of the eGFP gene in the P/M intergenic region, we replaced the exogenous gene expression cassettes in other IG (N/P, M/F, F/SH, SH/HN, and HN/L) with the P/M cassette. The gene expression cassette is composed of GE, IG, and GS signal sequences arranged in sequence at the insertion sites of exogenous genes (Fig. 6A). We constructed five types of rPIV5-JS17 using this P/M cassette (rPIV5-JS17-rPM): JS17NP-PMGFP, JS17MF-PMGFP, JS17FSH-PMGFP, JS17SHHN-PMGFP, and JS17HNL-PMGFP (Fig. 6B). These plasmids were co-transfected with pCAGGS-N, pCAGGS-P, pCAGGS-L, and pCAGGS-T7opt into BHK-21 cells. Daily monitoring of eGFP fluorescence (Fig. 6C) and harvesting of the culture medium after 7 days allowed for further analysis. Next, the cultures were expanded using Vero E6 cells, and virus particles expressing eGFP fluorescence from five types of rPIV5-JS17-rPM were successfully generated. We recorded eGFP fluorescence at 1, 3, and 5 dpi (Fig. 7A) and quantified the fluorescence intensity using Evos Analysis software. At both 1 and 5 dpi, the eGFP fluorescence intensity followed the order JS17NP-PMGFP > JS17SHHN-PMGFP > JS17MF-PMGFP > JS17FSH-PMGFP > JS17HNL-PMGFP (Fig. 7B). At 3 dpi, the order was JS17NP-PMGFP > JS17SHHN-PMGFP > JS17FSH-PMGFP > JS17MF-PMGFP > JS17HNL-PMGFP (Fig. 7B).

Additionally, the mRNA ratios of eGFP relative to PIV5-M and the protein levels of eGFP, PIV5-P, and PIV5-V verified that the expression of the exogenous gene JS17NP-PMGFP is the best (Fig. 7C and D). While the expression of exogenous genes in JS17NP-PMGFP surpassed that in JS17-NPGFP at 1 and 3 dpi, it lagged behind at 5 dpi (Fig. 7E). It is worth noting that the P/M cassette is a potent but conditionally dependent regulatory element. Its influence on gene expression is modulated by both the specific genomic integration site and the time post-infection (Fig. 7E). All five types of rPIV5-JS17-rPM exhibit similar growth kinetics (Fig. 7F).

### The safety profile of JS17-PMGFP virus was evaluated in swine

**Fig 8:**
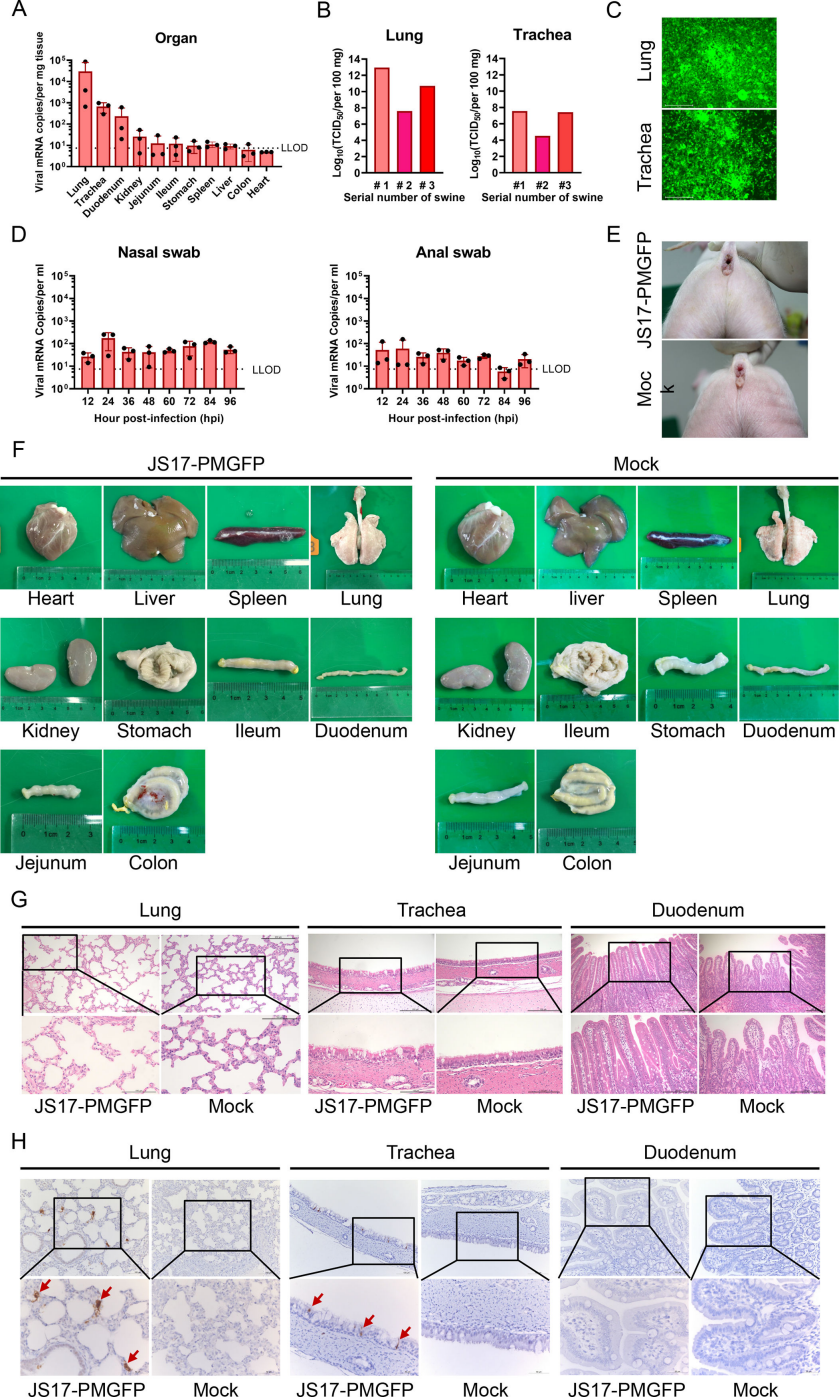
Establishment of an oral JS17-PMGFP infection model in swine. (**A**) Four days post-infection, viral nucleic acid loads in piglet organs were quantified by RT-qPCR. The dashed line indicates the lower limit of detection (LLOD). (**B**) Live virus loads in the lungs and trachea were determined by TCID_50_ assay. (**C**) Viruses isolated from the lungs and trachea can stably express eGFP fluorescence *in vitro* (**D**) Nasal and rectal swabs were collected every 12 h post-infection, and nucleic acid was detected by RT-qPCR. (**E**) Clinical diarrhea symptoms in the infection group and the control group. (**F**) Morphological changes in heart, liver, spleen, lungs, trachea, kidneys, stomach, duodenum, jejunum, ileum, and colon. (**G**) Pathological conditions in lungs, airways, and duodenum were examined. The upper box highlights regions analyzed at higher magnification in the lower box. (**H**) Immunohistochemical staining was performed for PIV5-P/V protein.

Since PIV5-JS17 was isolated from the intestine of pigs, we wondered whether rescued JS17-PMGFP could infect intestinal epithelial cells by oral route. To test this possibility, we chose 3-day-old piglets to determine the delivery efficiency of PIV5-JS17 by using JS17-PMGFP (Fig. S3). The results showed that at 4 days post-infection (dpi), recombinant viruses can only be detected in the lungs, trachea, and duodenum (Fig. 8A and B). Due to contamination from feces and pollutants, the virus titer in the duodenum was undetectable. Next, we isolated JS17-PMGFP from infected lungs and tracheae and inoculated it into Vero E6 cells. The results showed that viruses isolated from animals can infect naïve cells in culture (Fig. 8C). Further, the eGFP protein in the lungs infected with JS17-PMGFP was confirmed through immunohistochemistry (Fig. S4). This confirmed its effective infection in piglets and the successful expression of exogenous proteins. Low-level virus shedding was detected in nasal and anal swabs (Fig. 8D). Notably, no significant symptoms, such as diarrhea (Fig. 8E) or respiratory issues, were observed post-challenge with JS17-PMGFP.

Morphologically, all tissues exhibited no significant changes (Fig. 8F). Then, we performed RT-qPCR to analyze the expression of key cytokines in infected piglets. Among the Th1-related cytokines analyzed (IFN-γ, TNF-α), only IFN-γ showed a significant increase. For Th2-related cytokines (IL-4, IL-5, IL-13, IL-10), no significant changes were observed. Regarding inflammatory markers (IL-18, IL-6, IL-1β), only IL-1β was significantly elevated, while IL-6 and IL-18 remained unchanged. The results revealed a specific and limited host immune response (Fig. S5). To assess pathological alterations, H&E staining was performed on lung, trachea, and duodenum tissues. No widespread or severe pathological lesions were observed in the duodenum (Fig. 8G). Compared with the control group, the trachea of infected piglets exhibited fibrous tissue hyperplasia in the submucosal layer, and the lungs of JS17-PMGFP-infected piglets showed mildly widened alveolar septa with minimal inflammatory cell infiltration, consistent with a localized immune response to viral replication (Fig. S6). All animals remained clinically healthy, supporting the conclusion that the vector is safe in 3-day-old piglets. Immunohistochemical analysis confirmed the presence of PIV5 antigen in the lungs and trachea (Fig. 8H). In summary, we successfully established an oral infection model for piglets with JS17-PMGFP, demonstrating no significant pathogenic effects.

### JS17-PDCoV-S can mediate the production of neutralizing antibodies and cellular immunity in piglets

**Fig 9:**
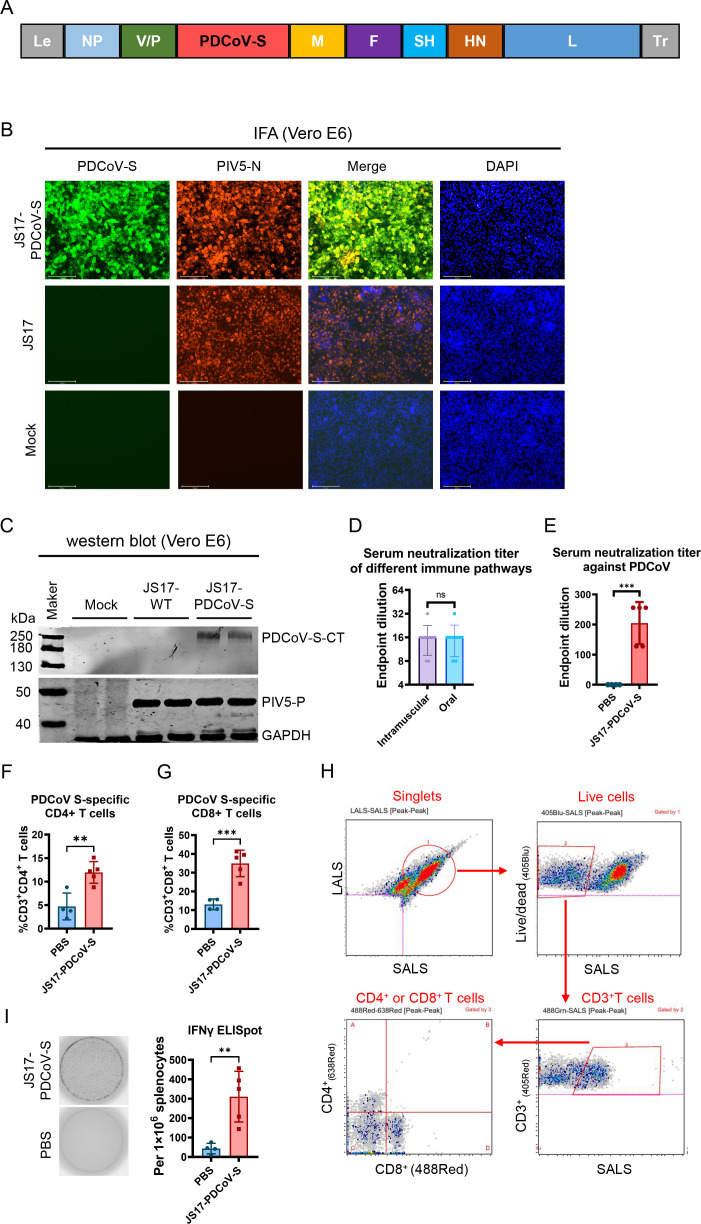
JS17-PDCoV-S can mediate the production of neutralizing antibodies and cellular immunity in piglets. (**A**) Schematic of JS17-PDCoV-S. Le, leader sequence; NP, nucleoprotein; V, V protein; P, phosphoprotein; M, matrix protein; F, fusion protein; SH, small hydrophobic protein; HN, hemagglutinin-neuraminidase protein; L, RNA-dependent RNA polymerase; Tr, trailer sequence. (**B**) IFA analysis of Vero E6 cells infected with JS17-WT and JS17-PDCoV-S. (**C**) Western blotting identification of JS17-PDCoV-S. (**D**) Neutralizing antibody titers via intramuscular injection and oral route. (**E**) Neutralizing antibody titers of JS17-PDCoV-S immunized piglets via oral route. (**F**) Using purified PDCoV-S to stimulate JS17-PDCoV-S immunized peripheral blood lymphocytes of pigs for 18 h, CD3^+^ and CD4^+^ T cells were detected by flow cytometry. (**G**) Flow cytometry detection of CD3^+^ and CD8^+^ T cells. (**H**) The flow cytometry grouping scheme was used to analyze the data using Apogee Flow cytometry analysis software (v6.0.96) (**P < 0.01; ***P < 0.001; ns, not significant). (**I**) IFN-γ ELISpot assays.

To investigate the capacity of the PIV5-JS17 vector for heterologous protein expression and the potential of such proteins to elicit immune responses in piglets, we utilized the reverse genetic system of JS17-PMGFP to insert the PDCoV S gene into the P/M intergenic region, replacing the GFP gene and generating JS17-PDCoV-S (Fig. 9A). Successful expression of the PDCoV S protein from the PIV5-JS17 vector was confirmed by IFA and Western blot (Fig. 9B and C).

To identify the optimal immunization route, we compared neutralizing antibody levels following oral and intramuscular administration. The results indicated no significant difference between the two routes (Fig. 9D). Subsequently, piglets were immunized orally with a prime-boost regimen at a 14-day interval, receiving a total dose of 1 × 10^5^ TCID_50_ in 5 mL per piglet. Compared to the PBS control group, the JS17-PDCoV-S immunized group exhibited neutralizing antibody titers ranging from 1:128 to 1:256 (Fig. 9E). Additionally, lymphocytes were isolated from piglet blood and stimulated with PDCoV S protein. The JS17-PDCoV-S group showed a significant induction of PDCoV S-specific CD4^+^ and CD8^+^ T cell responses compared to the PBS control (Fig. 9F through H). Furthermore, IFN-γ ELISpot assays revealed that S protein-specific T cells displayed a pronounced Th1-type polarization (Fig. 9I).

## DISCUSSION

There is a pressing need for novel oral vector vaccines to combat porcine diseases. Administering vaccines orally can significantly reduce pig stress during the immunization process, thereby potentially decreasing the infection and transmission of infectious diseases by stimulating mucosal immunity (29). This approach also aligns with the fundamental principles of animal welfare. In this study, we utilized a reverse genetic system based on a novel porcine PIV5 isolated from swine intestines. By using eGFP as a model for exogenous gene, we found that the P/M intergenic region is the best site for inserting exogenous genes.

The PIV5-W3A strain, the first discovered and studied, is considered a classic strain (30). It uniquely originates from monkeys, with no other PIV5 strains identified as having a monkey origin, suggesting that monkeys may not be the natural host. The PIV5-HLJ strain is genetically similar to the JS17 strain, differing only in the HN and L genes (Table S1). Notably, PIV5-HLJ forms typical CPE *in vitro*, while the JS17 strain does not, potentially due to differences in the HN or L gene (23). Additionally, there are significant growth kinetics differences between these two strains, with PIV5-HLJ reaching the highest virus titer at 3 dpi, while the JS17 strain reached it at 5 dpi (23). Previous studies linked CPE production to the P and F genes, but our analysis suggests potential novel mechanisms for future research (28, 31).

In prior research, the PIV5 reverse genetic system was constructed utilizing the classic monkey-derived PIV5-W3A strain, enhancing exogenous gene expression at the HN/L site by substituting the original cassette with an N/P cassette (30). Furthermore, through a comparative analysis of the insertion sites M/F, F/SHN, SH/HN, and HN/L, it has been conclusively demonstrated that the F/SHN and SH/HN insertion sites are superior to the HN/L insertion site (32). Currently, the intergenic region of SH/HN is commonly used for PIV5 insertions, including the spike protein of SARS-CoV-2, the spike protein of MERS, and the hemagglutinin (HA) protein from the influenza virus (17, 24, 25). Other paramyxovirus vectors, such as avian metapneumovirus and NDV, use the P/M site optimally (26, 33). Our research on the porcine-derived PIV5-JS17 strain contrasts with PIV5-W3A findings, showing the P/M intergenic region as the optimal insertion site for exogenous genes. Previous studies have shown that the mRNA of the virus gradually diminishes from the 3′ end to the 5′ end of its genome (12). However, our findings reveal that the expression level of the exogenous gene eGFP in the P/M intergenic region surpasses that of its upstream N/P intergenic region. Moreover, the expression level of eGFP in the F/SH gene intergenic region also surpasses that in its upstream M/F gene intergenic region. Mechanistic exploration revealed that exogenous gene expression is influenced by both the insertion site and the expression cassette. It implies that the insertion of exogenous genes does not align with the transcription polarity mechanism of PIV5, but rather is influenced by two factors: the location of insertion and the exogenous gene expression cassette. Therefore, selecting appropriate insertion sites and exogenous gene expression cassettes is pivotal, which may also apply to other paramyxoviruses used as gene delivery vectors. Our findings serve as a theoretical foundation or a valuable reference for studying PIV5 and other paramyxovirus gene delivery vectors.

Prior research has indicated the absence of PIV5 viral nucleic acid in porcine lungs sacrificed at 7 dpi (34). We evaluated the safety of JS17-PMGFP in swine and found that viral nucleic acids were detected in the lungs, trachea, and duodenum at 4 dpi. While the parental PIV5-JS17 strain was originally isolated from the porcine gastrointestinal tract, our experimental infection in piglets demonstrated a clear preference for efficient replication in the lung. Although the original isolation site was the intestine, this may not strictly define the virus’s optimal replication niche *in vivo*. Viral entry and replication efficiency are governed by complex interactions with host factors, such as local receptor availability, mucosal barriers, and the protease environment in specific tissues. It is possible that the cellular and microenvironmental conditions in the porcine respiratory epithelium are more favorable for PIV5 replication. Regarding the presence of the virus in the GI tract, this is likely a route through which viral particles reach the intestine following oral entry and swallowing. From an applied perspective, this observed lung tropism is not a limitation but rather a defining and advantageous feature for the development of PIV5-JS17 as a mucosal vaccine vector. The respiratory tract, particularly the lung, serves as a critical primary barrier and immune induction site for a multitude of airborne pathogens. The ability of our vector to target this tissue efficiently and elicit a localized immune response, as evidenced by the mild inflammatory cell infiltration in alveolar septa, aligns with the established paradigm for intranasal PIV5-based vaccines, which are renowned for their capacity to stimulate robust mucosal and systemic immunity. Most importantly, this targeted engagement of the lung mucosa can have far-reaching protective consequences. Due to the functional integration of the common mucosal immune system, immune effector cells (e.g., IgA-secreting B cells and tissue-resident memory T cells) primed in the lung can disseminate, providing surveillance at distant mucosal sites, including the gastrointestinal and urogenital tracts. Therefore, a vaccine delivered via this PIV5-JS17 vector platform holds substantial promise not only for preventing primary respiratory infections but also for potentially offering cross-mucosal protection against enteric pathogens, a hypothesis that warrants dedicated future investigation. This dual potential significantly broadens the scope and appeal of PIV5-JS17 as a versatile tool for porcine vaccine development.

Based on the PIV5-JS17 vector, we successfully constructed JS17-PDCoV-S, which expresses the PDCoV S protein. Through oral administration, we mediated the production of PDCoV-neutralizing antibodies and PDCoV S-specific T cell immunity in piglets. This further demonstrates the ability of PIV5-JS17 vector to deliver exogenous genes and its potential as a vector vaccine for pigs.

In conclusion, we have determined the optimal exogenous gene insertion site for PIV5-JS17, a novel porcine-derived strain, and clarified the mechanisms affecting exogenous gene expression. The swine infection model, established via oral administration, avoids the need for anesthesia during nasal challenge. Preliminary evidence confirms the safety of rPIV5-JS17 in swine and the immunogenicity of JS17-PDCoV-S in piglets, providing valuable insights and laying a solid foundation for future development of porcine-derived PIV5-based vector vaccines.

## MATERIALS AND METHODS

### Cell lines and plasmids

BHK-21 (baby hamster kidney fibroblasts) cells and Vero E6 (ATCC CRL-1586) cells were maintained in Dulbecco’s modified Eagle medium (DMEM) (Gibco) containing 10% fetal bovine serum (FBS). All cell lines were maintained in a humidified environment containing 5% CO_2_ at 37°C, with regular checks for mycoplasma contamination. The POK12 and pCAGGS plasmids were stored in our laboratory.

### Isolation of the JS17 strain of PIV5

Intestinal samples were collected from diarrheal piglets in Jiangsu Province, China. In brief, piglet intestinal tissues were ground in liquid nitrogen, and 200 μL of PBS was added per 30 mg of ground sample. After vigorous shaking, the supernatant was centrifuged at 8,000 × *g* for 15 min and filtered through a 0.22 μm filter (Merck Millipore). The filtrate was then mixed 1:3 with DMEM containing 5% penicillin, gentamicin, and streptomycin (Procell, China). This mixture was added to T25 culture flasks containing a monolayer of Vero E6 cells. After 2 h, the mixture was removed, and the cells were washed twice with DMEM. DMEM containing 1% penicillin, gentamicin, and streptomycin was then added, and the cells were cultured for 4 days. The culture was blindly passaged for three generations and stored at −80°C.

### Immunofluorescence assay (IFA)

IFA was employed to detect viral infection. On the fourth dpi, the cells were fixed with 4% paraformaldehyde at 4°C for 30 min. Following fixation, the cells were permeabilized with 0.1%Triton X-100 at room temperature for 15 min. The cells were then blocked with 5% skimmed milk. A monoclonal antibody, prepared in our laboratory, was incubated with the cells at 37°C for 2 h to target the PIV5-N or PIV5-P/V (Biodragon, China). Subsequently, a goat anti-mouse IgG (H+L) cross-adsorbed secondary antibody with Alexa Fluor 546 (Thermo Fisher Scientific) was used for 1 h at 37°C. The nuclei were stained with DAPI (Beyotime, China) for 15 min at room temperature. The cells were observed using an inverted fluorescence microscope (EVOS M5000, Life, USA).

### Electron microscopy

Four days post-virus infection, the cells were subjected to a single freeze-thaw cycle at −80°C. Cell debris was removed by centrifugation at 5,000 × *g* for 15 min at 4°C using an F-34-6-38 rotor in an Eppendorf Centrifuge 5804R. The supernatant was then collected and centrifuged at 100,000 × *g* for 2.5 h at 4°C. The sediment was collected and resuspended in 1 mL of DMEM per tube, followed by centrifugation. The sediment was collected and resuspended in 1 mL of DMEM per tube, followed by centrifugation through 13 mL 40–60% (wt/vol) sucrose cushion, at 100,000 × g for 2.5 h at 4°C. White bands formed by virions at the interface of sucrose solution were collected, and the sucrose was eluted with 70 mL of PBS for 2.5 h. The purified virus was then deposited onto a copper mesh, stained with phosphotungstic acid for 5–10 s, and observed under TEM (Hitachi H-7650).

### RNA extraction and full-length PCR amplification of PIV5-JS17

RNA was extracted from 140 μL of viral medium using the QIAamp Viral RNA Mini Kit (QIAGEN) following the manufacturer’s instructions. The RNA was eluted in 30 μL of RNase-free and DNase-free water and used as the template for cDNA synthesis with PrimeScript II 1st Strand cDNA Synthesis Kit (TAKARA). To determine the 5′ and 3′ ends of the genome, 5′ and 3′ RACE was performed using the SMARTer RACE 5′/3′ Kit (Takara). The primers used for sequencing the PIV5-JS17 strain are listed in Table S2, and the RACE primers are listed in Table S3.

### Development and screening of PIV5-N specific monoclonal antibodies

Six-week-old female BALB/c mice were immunized subcutaneously with 100 μg of purified virions inactivated by β-propanolactone (33672.51; SERVA, Germany) every 2 weeks for a total of four immunizations. Freund’s complete adjuvant (F5881; Sigma-Aldrich) was used for the first immunization, and Freund’s incomplete adjuvant (F5506; Sigma-Aldrich) was used for the remaining three. After the fourth immunization, mice received a booster immunization of 2 μg of purified and inactivated virions via tail vein injection. Four days later, mice were euthanized, and spleens were collected to prepare splenocyte suspensions after removing red blood cells using lymphocyte isolate (P8860; Solarbio, China). Splenocytes were isolated and fused with S/P20 cells according to standard procedures (35) and seeded in 96-well plates with medium containing 20% FBS and 1×Hybri-Max HAT medium supplement (H0262-10VL; Sigma). After 7 days, the medium was replaced with 20% FBS and 1×Hybri-Max HT (H0137-10VL; Sigma) supplement. Antibodies secreted by the fused cells in the culture medium supernatant were identified by PIV5-N indirect ELISA. Three rounds of limited dilution subcloning were carried out on positive hybridoma to obtain purified hybridoma cell lines.

### Immunoblotting

As described in previous studies (20), in brief, the membrane was blocked with 5% skim milk and incubated with primary antibodies against PIV5-P/V protein (Biodragon), PIV5-N, GFP (SinoBiological), and glyceraldehyde-3-phosphate dehydrogenase (GAPDH) (Thermo Fisher Scientific). IRDye 800-conjugated goat anti-mouse IgG (Thermo Fisher Scientific) was used as the secondary antibody. The membrane was scanned and analyzed with an Odyssey infrared scanner (Li-Cor Biosciences).

### Nucleotide sequence similarity analysis and phylogenetic analysis of the JS17 strain of PIV5

SimPlot software was used to analyze the similarity of PIV5 strains from various species (36). Editseq software was used for sequence manipulation and storage. Multi-sequence alignment was performed using MUSCLE in MEGA X (10.1.5) with default parameters. Whole-genome sequence analysis was conducted using the maximum likelihood method and a neighbor-joining method with the general time reversible (GTR) model, gamma-distributed rates with invariant sites (G+I), and 1,000 bootstrap replicates. For the F genome, the Tamura 3-parameter model (T92) with uniform rates and 1,000 bootstrap replicates was used. For the HN genome, the T92 model with gamma-distributed (G) rates and 1,000 bootstrap replicates was employed. SimPlot software was used for whole-genome similarity analysis with default parameters.

### Plasmid construction

As described in previous studies (20), in brief, the IG N/P, P/M, M/F, F/SH, SH/HN, and HN/L were inserted into a single BmtI restriction site, with eGFP inserted into these sites to create the JS17-NPGFP, JS17-PMGFP, JS17-MFGFP, JS17-FSHGFP, JS17-SHHNGFP, and JS17-HNLGFP plasmids. Regarding JS17-PDCoV-S, the S gene of PDCoV NH strain, after codon optimization in mammalian cells, was inserted into the P/M intergenic region (37).

### Rescue of rPIV5-JS17 strain

To rescue the infectious recombinant PIV5, the plasmids JS17-NPGFP, JS17-PMGFP, JS17-MFGFP, JS17-FSHGFP, JS17-SHHNGFP, JS17-HNLGFP, and JS17-PDCoV-S, along with the helper plasmids pCAGGS-N, pCAGGS-P, and pCAGGS-L, were co-transfected into BHK-21 cells at 90% confluence using JetOPTIMUS (Polyplus). The ratio of the number of plasmids per well in a 12-well cell culture plate was as follows: 1.5 μg of each full-length genome plasmids (JS17-NPGFP, JS17-PMGFP, JS17-MFGFP, JS17-FSHGFP, JS17-SHHNGFP, JS17-HNLGFP), 0.5 μg of pCAGGS-N, 0.15 μg of pCAGGS-P, 0.75 μg of pCAGGS-L, and 1 μg pCAGGS-T7opt (17). After mixing the plasmids with jetOPTIMUS buffer, 2.9 μL of jetOPTIMUS DNA transfection reagent was added and incubated for 10 min at room temperature. The cells were cultured at 37°C, with half of the medium replaced on day 4 post-transfection. The culture was collected and stored at −80°C on day 7.

### Growth kinetics

As previously described, Vero E6 cells were infected with PIV5 or rPIV5-JS17 at an MOI of 0.1 (24). After 1 h of adsorption, DMEM supplemented with 2% FBS was added to the plate. Supernatant samples were collected daily for five consecutive days and stored at −80°C.

### RT–qPCR for different rPIV5-JS17 and organs and swabs of swine

RNA was extracted from Vero E6 cells infected with various strains of rPIV5-JS17 (MOI = 1) at 1, 3, and 5 dpi, as well as from organs and swabs of swine and mice, using the Simply P Total RNA Extraction Kit (BioFlux). cDNA was synthesized using the PrimeScript 1st Strand cDNA Synthesis Kit (TAKARA). For the relative quantitation of rPIV5-JS17 in infected Vero E6 cells, the TB Green Premix Ex Taq Tli RNaseH Plus ROX plus (TAKARA) was employed. The Ex Taq-Probe qPCR (TAKARA) was used for absolute quantitation of virus RNA copy number in the organs and swabs of swine and mice. RT-qPCR was performed on the QuantStudio 5 Real-Time PCR System. The primers used for relative quantitation, specific to GFP, PIV5-M, and GAPDH, are detailed in Table S4. The expression levels of eGFP and PIV5-M were normalized to GAPDH expression. Relative gene expression was calculated using the 2^−ΔΔCt^ method. The primers used for absolute quantitation are listed in Table S5. Based on prior research, the limit of detection (LLOD) was established with a Ct value of 35, corresponding to a copy number of 7.4 copies per mL.

### Animal infection experiments

Six 3-day-old piglets were divided into two groups, with three animals per group. One group was orally challenged with 5 mL of JS17-PMGFP strain, with a virus titer of 1×10^5^ TCID_50_. The other group served as the control and received 5 mL of PBS orally. Following the challenge, anal and nasal swabs were collected and tested at 12-hour intervals, and the viral RNA load in both swabs was quantitatively determined using RT-qPCR. Four days post-challenge, euthanasia was conducted, and morphological changes were documented in the heart, liver, spleen, lungs, trachea, kidneys, stomach, duodenum, jejunum, ileum, and colon. RT-qPCR was utilized to quantify the viral nucleic acid load across various tissues, while the Reed method was adopted to quantify the viable virus particle load in organs, expressed as TCID_50_/g. Additionally, specific organs underwent pathological and immunohistochemical analyses.

### Histopathological and immunohistochemistry assay

For histopathological analysis, the organ tissues from infected pigs or mice were fixed in 10% formalin overnight. Subsequently, the fixed samples were processed using an automated enclosed dehydrator (STP 420 D, Thermo Fisher Scientific) and then embedded in paraffin. The embedded tissues were then sliced to a thickness of 4 μm using an ultrathin microtome (UC-6, RMC, USA) and stained with hematoxylin and eosin following standard methods.

For immunohistochemistry assay, the swine organ sections were mounted and sealed using the Tissue-Tek Film Coverslipper System (Tissue-Tek Film, Japan), following the previously described procedures (38). Detection of the JS17-PMGFP antigen was achieved through the utilization of the V5 Tag Monoclonal Antibody (2F11F7, Invitrogen, USA). Both histopathological and immunohistochemical assays were conducted using an optical microscopy (Nikon Eclipse E100, Tokyo, Japan) for observation and image acquisition.

### Neutralization antibody assay

As previously described, piglets were immunized with JS17-PDCoV-S, and their serum was isolated and collected 28 days later (34). After serum inactivation at 56°C for 30 min, it was continuously diluted in a twofold gradient and thoroughly mixed with an equal volume of PDCoV. The mixture was then incubated at 37°C for 1 h. Then, the mixture was added to a 96-well plate containing monolayer cells and cultured at 37°C for 4 days. The neutralization result was calculated by detecting the N protein expression of PIV5 through IFA.

### Flow cytometry

At 28 days post-immunization, peripheral blood lymphocytes were isolated from piglets. The lymphocytes were stimulated for 18 h with a purified, His-tagged PDCoV-S protein expressed in HEK 293 cells (29). Following stimulation, cells were first stained with a live/dead cell discrimination dye (LIVE/DEAD Fixable kits, Invitrogen, #L34964). They were then fixed using a flow cytometry fixation buffer (IC Fixation Buffer, Invitrogen, #00-8222-49) and subsequently stained for surface markers with the following fluorochrome-conjugated antibodies: Alexa Fluor 647-conjugated mouse anti-pig CD3ɛ (BD Biosciences, #561475), FITC-conjugated mouse anti-pig CD8α (BD Biosciences, #551303), and PerCP-Cy5.5-conjugated mouse anti-pig CD4α (BD Biosciences, #561474). All staining procedures were performed strictly in accordance with the manufacturers’ protocols. Data acquisition was carried out on an Apogee Flow Systems instrument.

### IFN-γ ELISpot

The frequency of PDCoV-S-specific IFNγ-secreting lymphocytes in peripheral blood samples from immunized piglets was determined using an ELISpot Plus: Porcine Interferon-γ (HRP) kit (Mabtech, #3130-4HPW-2). Briefly, isolated peripheral blood lymphocytes were stimulated with PDCoV-S protein for 48 h, with each well containing at least 2 × 10^6^ cells. The plates were kept undisturbed during the incubation period to avoid affecting spot formation. All experimental procedures were performed according to the manufacturer’s instructions. Spots were counted using an AID EliSpot Reader Classic (Germany), and data were analyzed with EliSpot 7.0S software.

### Statistical analysis

The data in the figures represent the mean or mean ± standard deviation (SD). Statistical comparisons between different groups were performed by Student’s *t*-test using GraphPad Prism 9 (Version 9.3.1, GraphPad, San Diego, California, USA). The error bars represent SD. Differences were considered statistically significant at *P* < 0.05 (*P*-values are *P < 0.05; **P < 0.01; ***P < 0.001; ****P < 0.0001; ns, not significant).

## Data Availability

The complete genome data of PIV5-JS17 used in this study, including precise 5′ and 3′ ends, have been uploaded to GenBank with accession number OR888917.1.

## SUPPLEMENTAL MATERIAL

Supplemental material jvi.01858-25-s0001.docx Figures S1 to S6; Tables S1 to S5.

ASM does not own the copyrights to Supplemental Material that may be linked to, or accessed through, an article. The authors have granted ASM a non-exclusive, world-wide license to publish the Supplemental Material files. Please contact the corresponding author directly for reuse.
