# Supplementary material for: The newly developed porcine-origin parainfluenza virus PIV5-JS17 serves as an exogenous gene delivery system for swine
Source: J Virol. 2026 Jan 13;100(2):e01858-25. doi: 10.1128/jvi.01858-25 (PMC12911860; doi:10.1128/jvi.01858-25)
Supplement: Supplemental material — Figures S1 to S6; Tables S1 to S5. [file jvi.01858-25-s0001.docx]

**The newly developed porcine-origin parainfluenza virus PIV5-JS17 serves as an exogenous gene delivery system for swine**

Guangyi Cong ^1^, Huan Li ^1^, Liang Li^1^, Jianfei Chen ^1^, Fang Fu ^1^, Huiwei Deng ^1^, Zedong Hu ^1^, Linan Wang ^1^, Yijing Li*^,2^, Mei Xue*^,1^ , Li Feng*^,1^

1 State Key Laboratory for Animal Disease Control and Prevention, Harbin Veterinary Research Institute, Chinese Academy of Agricultural Sciences, Harbin, Heilongjiang, China

2 College of Veterinary Medicine, Northeast Agricultural University, Harbin, Heilongjiang, China

*Corresponding Authors, Mei Xue ([xuemei_23@126.com](mailto:xuemei_23@126.com)), Li Feng ([fengli_h@163.com](mailto:liupinghuang@caas.cn)) and yijingli@163.com

**
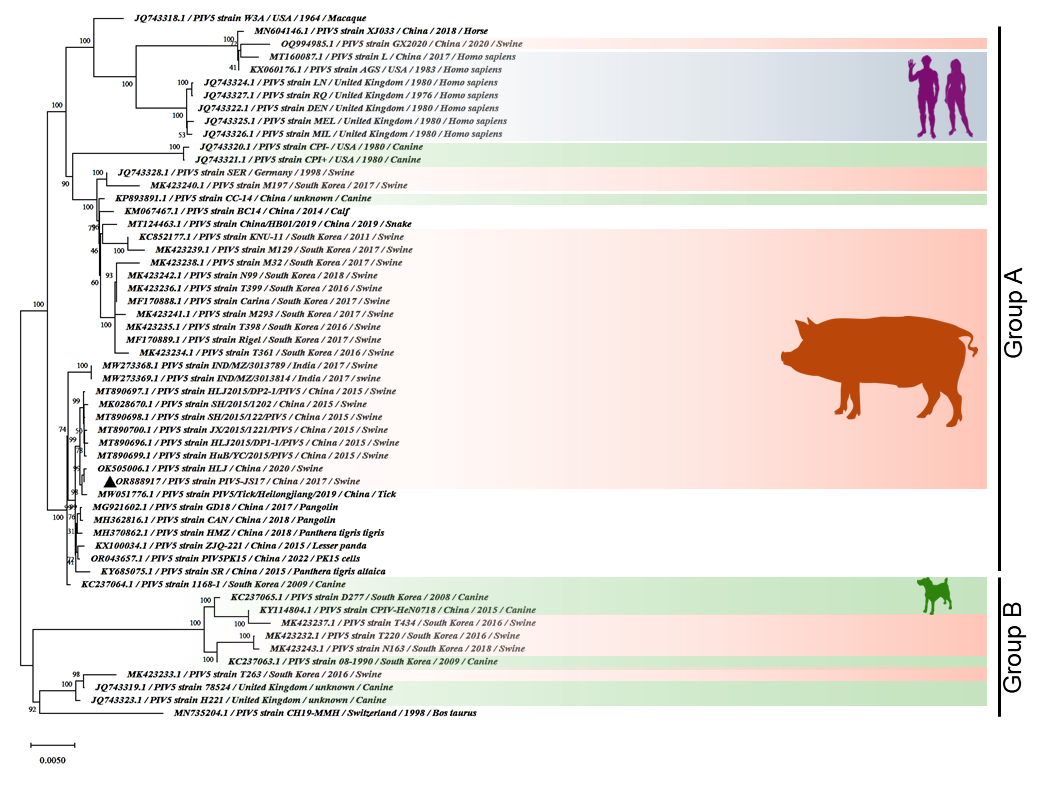
**

**Fig S1.** **A whole-gene phylogenetic analysis is based on PIV5 nucleotides.**

**
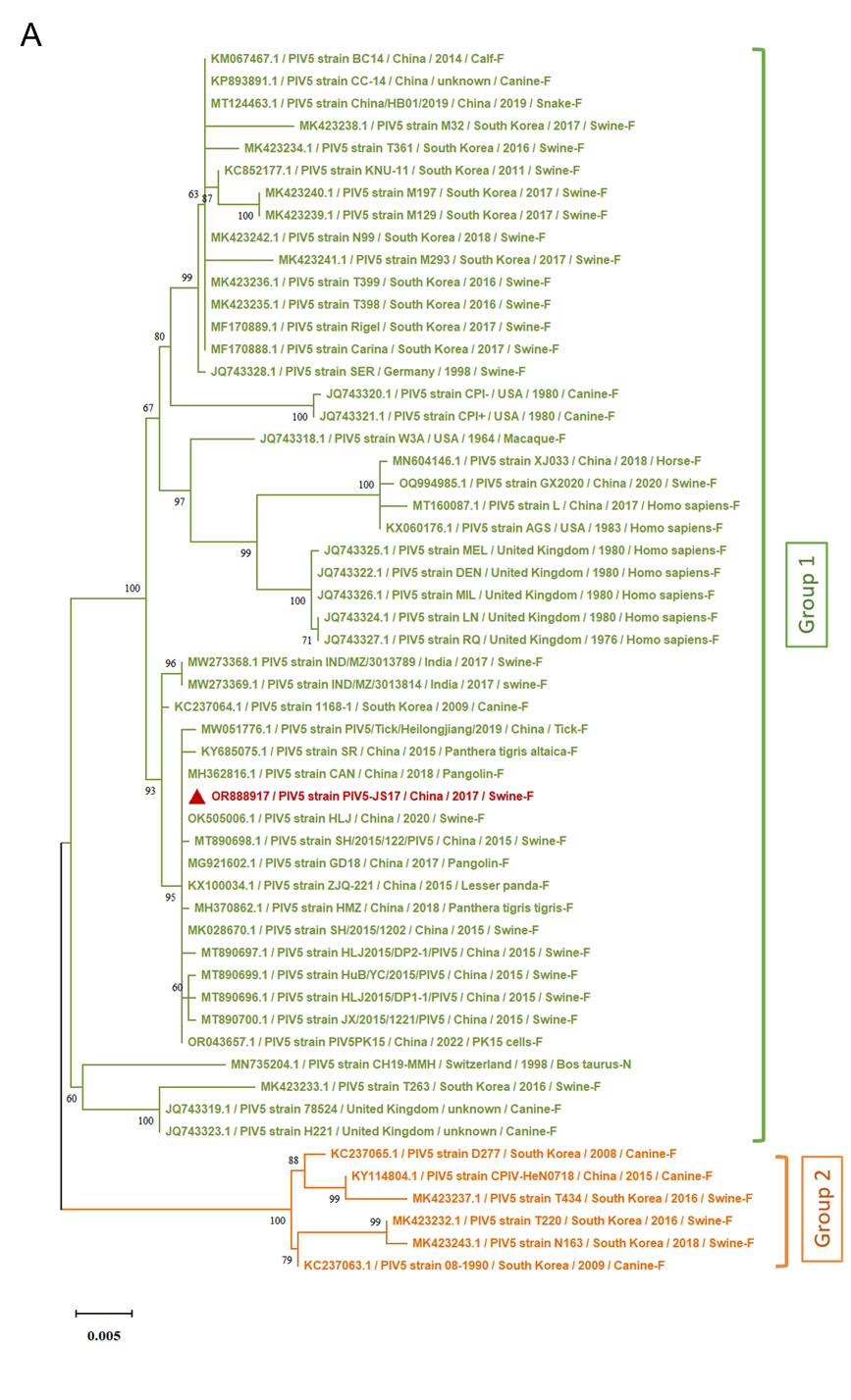
**

**
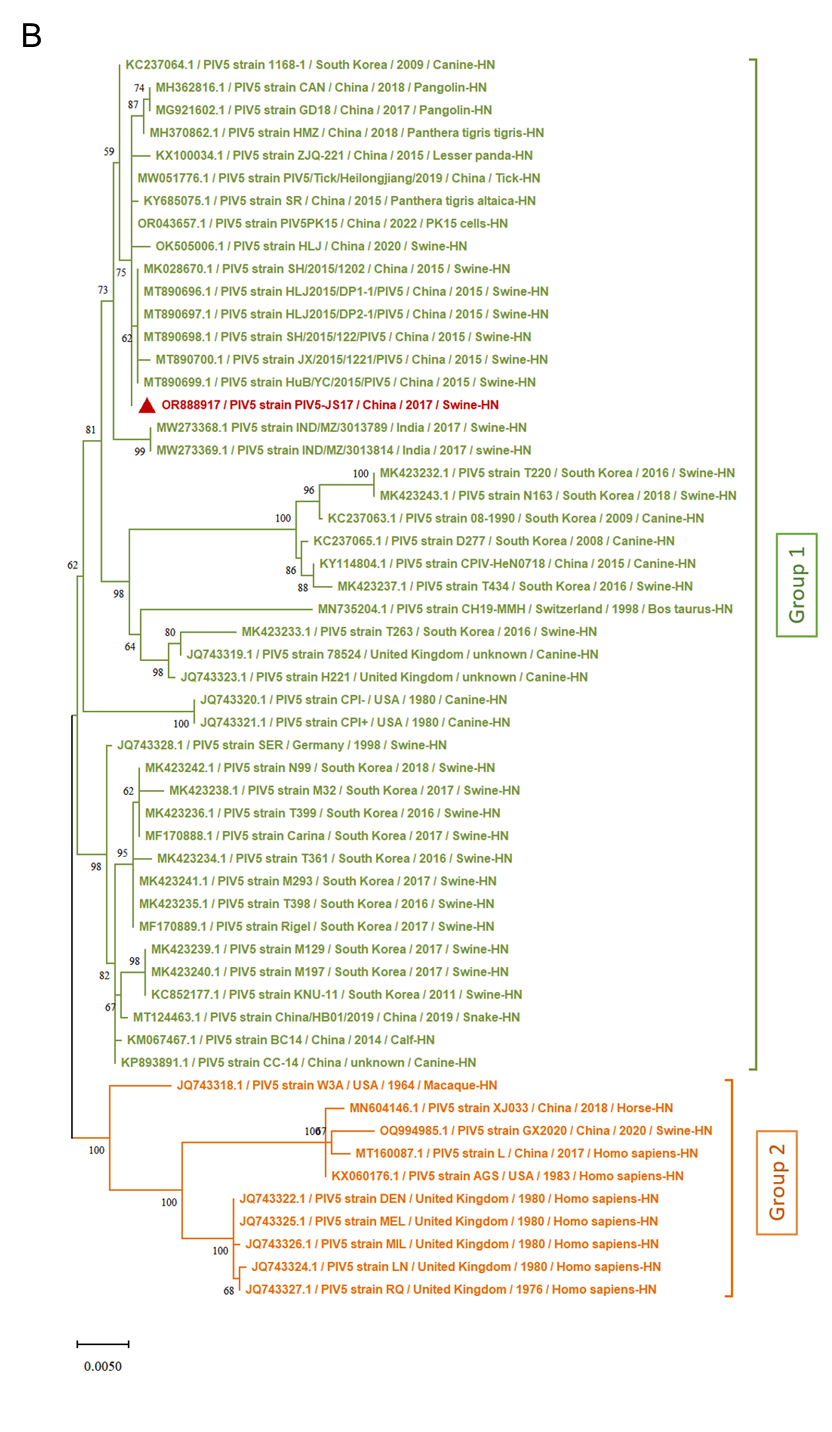
**

**Fig S2.** (A) A phylogenetic analysis specifically of the PIV5 F gene. (B) A phylogenetic analysis of the PIV5 HN gene. The red triangle denotes strains isolated in this study. The scale indicates the number of substitutions per site. The methodology section includes details on settings and software used.


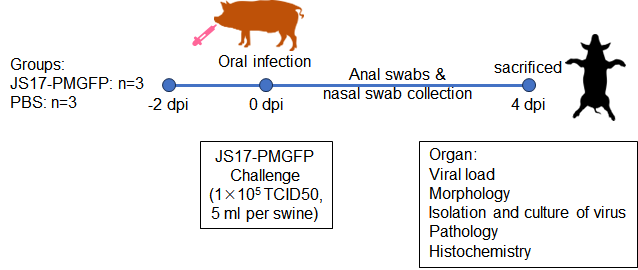


**Fig S3. Schematic timeline of PIV5-JS17 challenge in swine.** Swine were challenged with 5 ml of 1 × 10^5^ TCID_50_ PIV5-JS17, with PBS as a control. Nasal and rectal swabs were collected every 12 hours for viral nucleic acid load detection, and clinical symptoms such as diarrhea were observed. At 4 dpi, animals were euthanized for examination of histological morphology and viral nucleic acid load in various organs. The lungs, trachea, and duodenum were analyzed for pathology and immunohistochemistry. Additionally, the live virus load in the lungs and trachea was determined, and JS17-PMGFP expressing eGFP was isolated from these tissues.


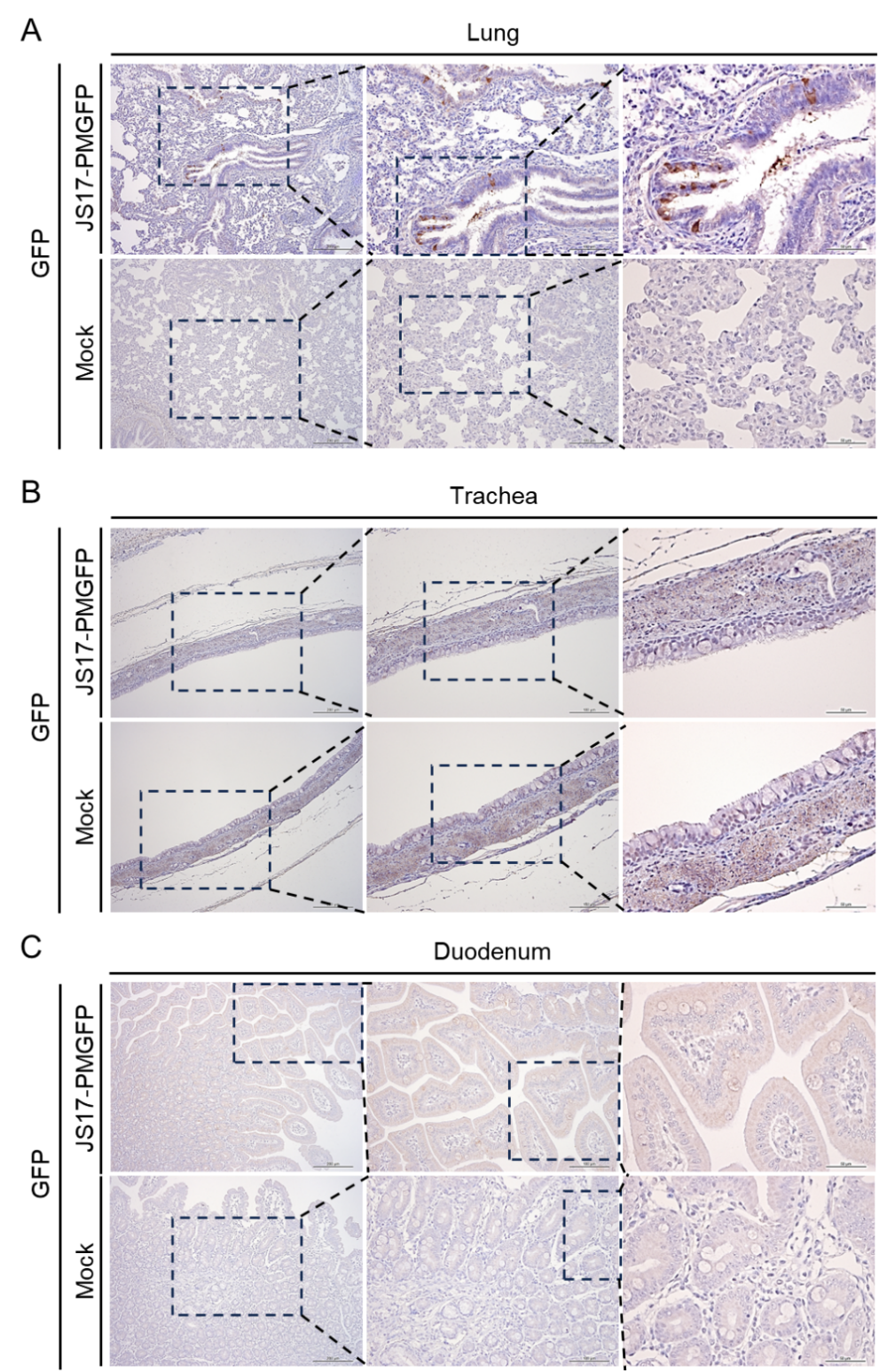


**Fig S4. The eGFP protein in the (A) lungs, (B) trachea, and (C) duodenum of piglets infected with JS17-PMGFP was detected through immunohistochemistry.**


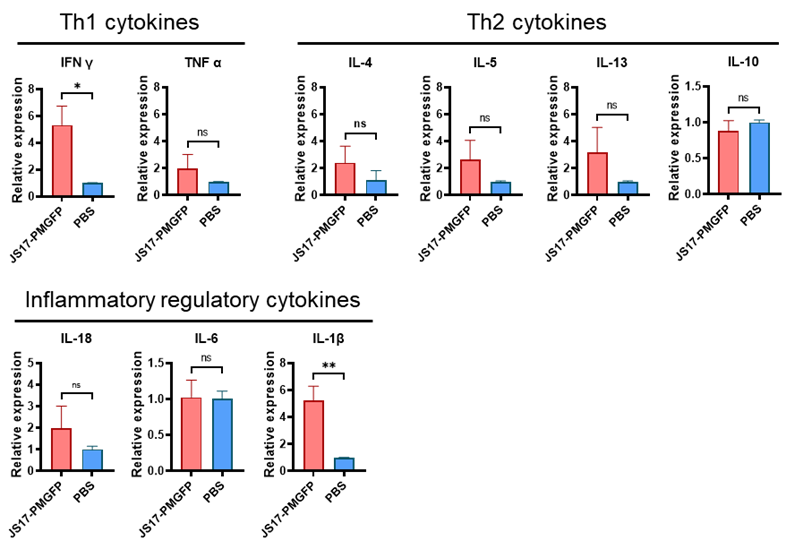


**Fig S5. Cytokine expression profile of piglets infected with JS17-PMGFP.** Th1 and Th2 cell related factors, cytokines, and inflammatory markers. The data presented represent the mean ± SD. Asterisks in the figures indicate significant differences (*p<0.05; **p<0.01 and ns, not significant).


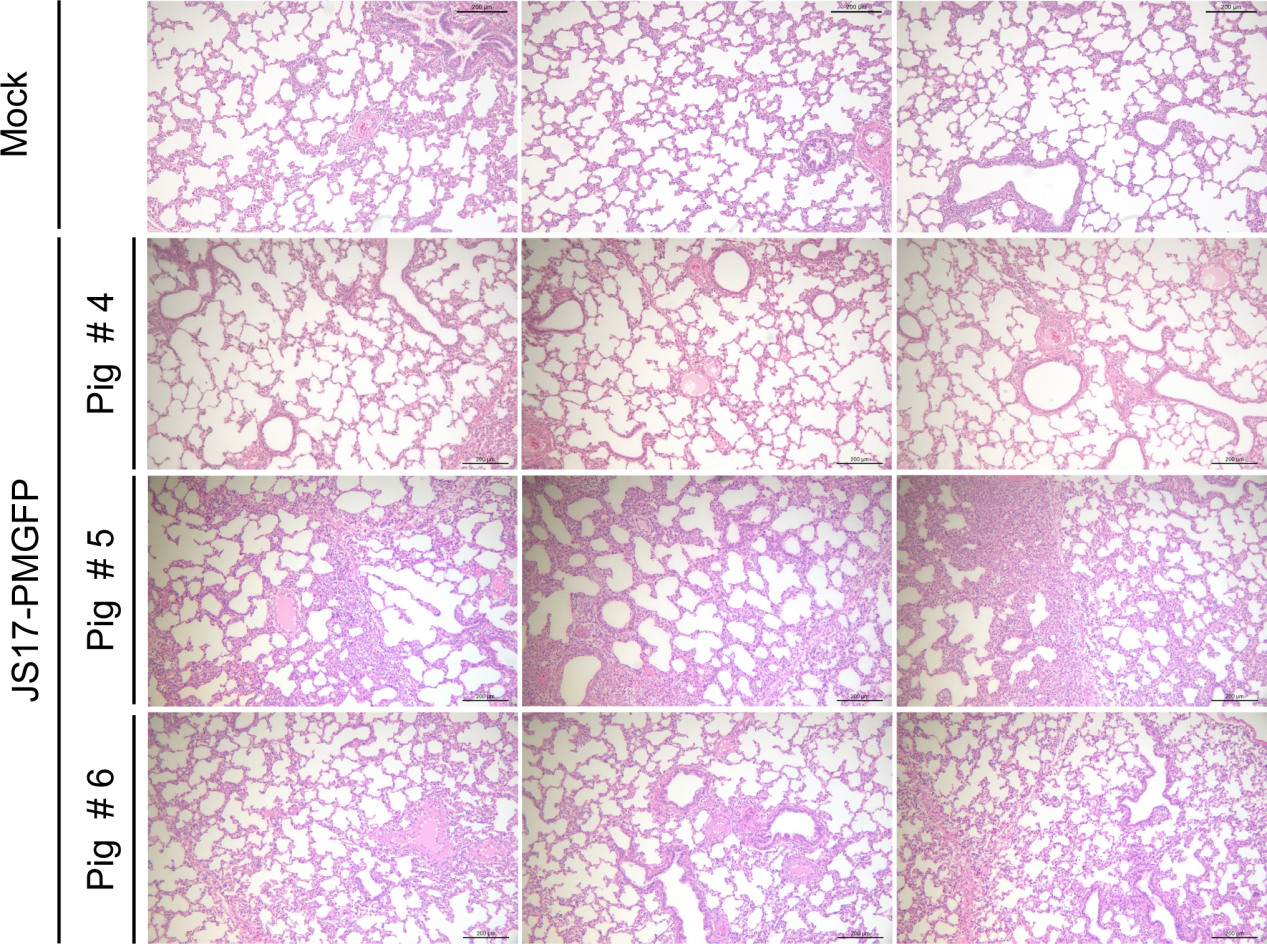


**Fig S6. Histopathology of lung tissue in piglets infected with JS17-PMGFP.**

.

Supplementary Table 1

|  | Sequence identities with PIV5-JS17 (nt / aa %) | | | | | | | | | |
| --- | --- | --- | --- | --- | --- | --- | --- | --- | --- | --- |
| No. | Strain  (GenBank, Country, Species) | Full-length genome | NP | P | V | M | F | SH | HN | L |
|  | HLJ  (OK505006.1, China, Swine) | 99.97 | 100.0/100.0 | 100.0/100.0 | 100.0/100.0 | 100.0/100.0 | 100.0/100.0 | 100.0/100.0 | 99.82/99.8 | 99.97/100.0 |
|  | PIV5/Tick/Heilongjiang/2019  (MW051776.1, China, Tick) | 99.91 | 100.0/100.0 | 99.83/99.7 | 99.85/100.0 | 100.0/100.0 | 99.88/99.6 | 100.0/100.0 | 100.0/100.0 | 99.96/100.0 |
|  | SH/2015/122/PIV5  (MT890698.1, China, Swine) | 99.89 | 99.87/99.8 | 99.58/98.7 | 99.70/99.1 | 100.0/100.0 | 99.94/99.8 | 100.0/100.0 | 99.94/100.0 | 99.97/100.0 |
|  | HLJ2015/DP2-1/PIV5  (MT890697.1, China, Swine) | 99.89 | 99.87/99.8 | 99.58/98.7 | 99.70/99.1 | 100.0/100.0 | 99.82/99.6 | 100.0/100.0 | 99.94/100.0 | 99.99/100.0 |
|  | SH/2015/1202  (MK028670.1, China, Swine) | 99.88 | 99.80/99.6 | 99.58/98.7 | 99.70/99.1 | 100.0/100.0 | 100.0/100.0 | 100.0/100.0 | 99.94/100.0 | 99.94/100.0 |
|  | Hub/YC/2015/PIV5  (MT890699.1, China, Swine) | 99.87 | 99.87/99.8 | 99.58/98.7 | 99.70/99.1 | 100.0/100.0 | 99.88/99.6 | 100.0/100.0 | 99.94/100.0 | 99.96/100.0 |
|  | JX/2015/1221/PIV5  (MT890700.1, China, Swine) | 99.86 | 99.87/99.8 | 99.58/98.7 | 99.70/99.1 | 100.0/100.0 | 99.88/99.6 | 100.0/100.0 | 99.82/100.0 | 99.96/100.0 |
|  | HLJ2015/DP1-1/PIV5  (MT890696.1, China, Swine) | 99.86 | 99.87/99.8 | 99.58/98.7 | 99.70/99.1 | 99.82/99.8 | 99.88/99.6 | 100.0/100.0 | 99.94/100.0 | 99.94/100.0 |
|  | PIV5PK15  (OR043657.1, China, PK15) | 99.84 | 99.80/99.6 | 99.83/99.5 | 99.85/99.6 | 99.91/99.9 | 100.0/100.0 | 100.0/100.0 | 100.0/100.0 | 99.91/99.9 |
|  | GD18  (MG921602.1, China, Pangolin) | 99.83 | 99.87/99.8 | 99.83/99.5 | 99.85/99.6 | 100.0/100.0 | 100.0/100.0 | 100.0/100.0 | 99.82/99.6 | 99.93/99.9 |
|  | CAN  (MH362816.1, China, Pangolin) | 99.82 | 99.87/99.8 | 99.83/99.5 | 99.85/99.6 | 100.0/100.0 | 100.0/100.0 | 100.0/100.0 | 99.82/99.6 | 99.88/99.7 |
|  | HMZ  (MH370862.1, China, Panthera tigris tigris) | 99.81 | 99.74/99.6 | 99.83/99.5 | 99.85/99.6 | 99.91/99.9 | 99.94/99.8 | 100.0/100.0 | 99.88/99.8 | 99.90/99.8 |
|  | ZJQ-221  (KX100034.1, China, Lesser panda) | 99.78 | 99.67/99.0 | 99.83/99.5 | 99.85/100.0 | 99.91/99.7 | 100.0/100.0 | 100.0/100.0 | 99.82/99.8 | 99.87/99.9 |
|  | 1168-1  (KC237064.1, South Korea, Canine) | 99.76 | 99.80/99.8 | 99.83/99.5 | 99.85/99.6 | 99.74/99.7 | 99.76/99.3 | 100.0/100.0 | 99.88/99.6 | 99.87/99.8 |
|  | SR  (KY685075.1, China, Panthera tigris altaica) | 99.72 | 99.61/99.2 | 99.41/98.5 | 99.25/98.2 | 99.56/99.6 | 99.88/100.0 | 100.0/100.0 | 99.94/99.8 | 99.88/99.8 |
|  | IND/MZ/3013814  (MW273369.1, India, swine) | 99.6 | 99.6/99.4 | 99.6/99.0 | 99.7/99.1 | 99.6/99.6 | 99.6/99.1 | 100.0/100.0 | 99.5/98.9 | 99.8/99.7 |
|  | IND/MZ/3013789  (MW273368.1, India, swine) | 99.57 | 99.61/99.4 | 99.58/99.0 | 99.70/99.1 | 99.56/99.6 | 99.64/99.1 | 100.0/100.0 | 99.47/98.9 | 99.78/99.7 |
|  | CC-14  (KP893891.1, China, Canine) | 98.98 | 99.41/99.4 | 99.07/98.5 | 98.65/98.2 | 98.59/98.6 | 99.15/98.6 | None | 99.35/98.8 | 99.35/99.4 |
|  | SER  (JQ743328.1, Germany, swine) | 98.96 | 99.48/99.4 | 99.07/98.5 | 98.65/98.2 | 98.68/98.7 | 99.15/98.6 | None | 99.35/98.9 | 99.32/99.3 |
|  | China/HB01/2019  (MT124463.1, China, Snake) | 98.89 | 99.41/99.4 | 98.81/97.7 | 98.21/96.9 | 98.50/98.5 | 99.15/98.6 | None | 99.23/98.6 | 99.35/99.4 |
|  | PIV5-BC14  (KM067467.1, China, Calf) | 98.88 | 99.22/99.2 | 98.90/98.0 | 98.51/97.8 | 98.68/98.7 | 99.15/98.6 | None | 99.29/98.6 | 99.26/99.3 |
|  | Rigel  (MF170889.1, South Korea, swine) | 98.88 | 99.15/99.4 | 98.98/98.2 | 98.51/97.8 | 98.41/98.4 | 99.15/98.6 | None | 99.18/98.6 | 99.28/99.3 |
|  | Carina  (MF170888.1, South Korea, swine) | 98.86 | 99.15/99.4 | 98.90/98.0 | 98.51/97.8 | 98.41/98.4 | 99.15/98.6 | None | 99.12/98.6 | 99.26/99.3 |
|  | T398  (MK423235.1, South Korea, swine) | 98.8 | 99.2/99.4 | 99.0/98.2 | 98.5/97.8 | 98.4/98.4 | 99.2/98.6 | None | 99.2/98.6 | 99.3/99.3 |
|  | T399  (MK423236.1, South Korea, swine) | 98.8 | 99.2/99.4 | 99.8/98.0 | 98.5/97.8 | 98.4/98.4 | 99.2/98.6 | None | 99.1/98.6 | 99.3/99.3 |
|  | N99  (MK423242.1, South Korea, swine) | 98.8 | 99.2/99.4 | 98.9/98.0 | 98.5/97.8 | 98.4/98.4 | 99.2/98.6 | None | 99.1/98.6 | 99.3/99.3 |
|  | M293  (MK423241.1, South Korea, swine) | 98.76 | 99.08/99.2 | 98.90/98.0 | 98.36/97.3 | 98.15/98.1 | 98.55/97.3 | None | 99.18/98.6 | 99.23/99.2 |
|  | W3A  (JQ743318.1, USA, Macaque) | 98.76 | 99.08/99.2 | 99.07/99.0 | 98.80/99.1 | 98.24/97.6 | 98.73/98.1 | 97.04/97.0 | 98.47/97.5 | 99.08/99.3 |
|  | T361  (MK423234.1, South Korea, swine) | 98.75 | 98.82/98.4 | 98.73/98.0 | 98.06/97.3 | 98.24/98.2 | 98.85/97.6 | None | 99.00/98.1 | 99.25/99.2 |
|  | KNU-11  (KC852177.1, South Korea, swine) | 98.73 | 99.09/98.4 | 98.56/97.4 | 98.06/96.9 | 98.32/98.3 | 99.03/98.4 | None | 99.06/98.4 | 99.20/99.1 |
|  | H221  (JQ743323.1, United Kingdom, Canine) | 98.66 | 98.56/99.0 | 99.07/98.5 | 98.95/99.1 | 98.68/98.7 | 98.25/98.4 | 97.04/97.0 | 99.00/98.9 | 99.01/99.4 |
|  | 78524  (JQ743319.1, United Kingdom, Canine) | 98.62 | 98.50/99.0 | 98.98/97.2 | 98.65/98.7 | 98.68/98.7 | 98.25/98.4 | 97.04/97.0 | 98.94/98.9 | 98.98/99.6 |
|  | M32  (MK423238.1, South Korea, swine) | 98.6 | 98.50/97.6 | 98.56/97.2 | 98.06/96.9 | 98.41/98.4 | 98.37/96.9 | None | 98.88/97.9 | 99.19/99.2 |
|  | M197  (MK423240.1, South Korea, swine) | 98.6 | 98.95/97.8 | 98.47/97.2 | 97.76/96.4 | 97.97/98.2 | 98.37/97.6 | None | 98.94/98.4 | 99.14/98.8 |
|  | M129  (MK423239.1, South Korea, swine) | 98.56 | 98.82/97.8 | 98.39/97.2 | 97.76/96.4 | 98.24/98.2 | 98.67/97.6 | None | 99.06/98.4 | 99.08/98.8 |
|  | T263  (MK423233.1, South Korea, swine) | 98.53 | 98.04/97.8 | 98.47/97.4 | 98.06/97.3 | 98.50/98.5 | 97.52/97.1 | 97.04/97.0 | 98.53/98.1 | 98.86/99.2 |
|  | CPI+  (JQ743321.1, USA, Canine) | 98.13 | 98.37/99.2 | 98.73/98.0 | 98.21/97.8 | 97.88/97.9 | 98.19/97.5 | None | 98.47/98.4 | 98.80/99.3 |
|  | CPI-  (JQ743320.1, USA, Canine) | 98.09 | 98.37/99.2 | 98.39/ | 97.61/96.4 | 97.88/97.9 | 98.25/97.6 | None | 98.47/98.4 | 98.77/99.2 |
|  | DEN  (JQ743322.1, United Kingdom, Homo sapiens) | 98.08 | 98.17/99.0 | 98.47/98.0 | 98.06/98.7 | 97.88/97.9 | 98.25/98.2 | 96.30/96.3 | 97.88/98.1 | 98.46/99.2 |
|  | MIL  (JQ743326.1, United Kingdom, Homo sapiens) | 98.03 | 97.91/98.6 | 98.39/97.7 | 97.91/98.2 | 97.88/97.9 | 98.25/98.2 | 96.30/96.3 | 97.82/97.9 | 98.45/99.2 |
|  | MEL  (JQ743325.1, United Kingdom, Homo sapiens) | 98.02 | 97.91/98.4 | 98.47/98.0 | 98.06/98.7 | 97.80/97.8 | 98.13/97.9 | 96.30/96.3 | 97.88/98.1 | 98.45/99.2 |
|  | RQ  (JQ743327.1, United Kingdom, Homo sapiens) | 98.01 | 98.04/98.6 | 98.39/97.7 | 97.91/98.2 | 97.71/97.7 | 98.19/98.0 | 96.30/96.3 | 97.82/97.9 | 98.48/99.2 |
|  | LN  (JQ743324.1, United Kingdom, Homo sapiens) | 98.00 | 98.04/98.6 | 98.39/ | 97.91/98.2 | 97.71/97.7 | 98.19/98.0 | 96.30/96.3 | 97.76/97.9 | 98.46/99.2 |
|  | CH19-MMH  (MN735204.1, Switzerland, Bos taurus) | 97.81 | 98.17/98.0 | 98.30/97.7 | 97.91/98.7 | 98.23/98.2 | 97.77/97.5 | 91.9/91.9 | 97.76/97.5 | 98.30/98.9 |
|  | AGS  (KX060176.1, USA, Homo sapiens) | 97.54 | 97.91/98.6 | 98.30/98.0 | 97.61/97.8 | 97.18/97.2 | 97.64/97.6 | None | 97.06/97.0 | 98.12/98.5 |
|  | XJ033  (MN604146.1, China, Horse) | 97.49 | 97.91/98.6 | 98.30/98.0 | 97.61/97.8 | 97.18/97.2 | 97.64/97.6 | None | 96.94/96.6 | 98.08/98.4 |
|  | L  (MT160087.1, China, Homo sapiens) | 97.36 | 97.71/98.4 | 98.13/97.4 | 97.31/96.9 | 97.09/97.1 | 97.40/97.1 | 91.7/91.7 | 96.82/96.5 | 97.98/98.1 |
|  | GX2020  (OQ994985.1, China, Swine) | 97.25 | 97.58/98.2 | 97.88/96.7 | 97.16/96.4 | 96.74/96.7 | 97.52/97.5 | None | 96.64/96.5 | 97.89/98.1 |
|  | 08-1990  (KC237063.1, South Korea, Canine) | 97.23 | 97.19/98.4 | 97.28/94.9 | 97.46/96.9 | 96.83/96.8 | 96.86/96.9 | 85.9/85.9 | 97.64/97.3 | 98.29/99.0 |
|  | D277  (KC237065.1, South Korea, Canine) | 97.19 | 97.06/98.4 | 97.20/94.6 | 97.31/96.9 | 97.00/97.0 | 96.62/96.4 | 85.2/85.2 | 97.76/97.5 | 98.27/99.1 |
|  | CPIV-HeN0718  (KY114804.1, China, Canine) | 96.96 | 96.86/98.6 | 96.86/94.1 | 96.86/96.4 | 96.21/96.2 | 96.44/96.2 | 84.4/84.4 | 97.70/97.5 | 98.09/99.0 |
|  | N163  (MK423243.1, South Korea, Swine) | 96.89 | 96.73/97.5 | 96.77/94.4 | 97.00/96.4 | 96.47/96.5 | 96.26/94.7 | None | 97.23/96.6 | 98.17/98.6 |
|  | T220  (MK423232.1, South Korea, Swine) | 96.89 | 96.80/97.5 | 96.77/94.4 | 97.00/96.4 | 96.47/96.5 | 96.20/94.9 | None | 97.23/96.6 | 98.17/98.6 |
|  | T434  (MK423237.1, South Korea, Swine) | 96.71 | 96.67/98.0 | 96.77/93.9 | 96.86/96.4 | 96.03/96.0 | 95.89/95.1 | None | 97.53/97.3 | 97.84/98.4 |

Supplementary Table 2. Primers used for whole genome sequencing of PIV5-JS17 strain

| **Names** | **Sequences (5**'**-3**'**)** | **Annealing temperature** | **Length of PCR products** |
| --- | --- | --- | --- |
| JS17-1-F | TCTGGAACTTATGGCCTTCGT | 55 ℃ | 1388 bp |
| JS17-1-R | TATCTCTGCCTCAGTCGATCCGCTA | 61 ℃ |  |
| JS17-2-F | TGGAAACTGCAAGAAAACAACAGG | 56 ℃ | 1364 bp |
| JS17-2-R | TAATCATTCCTTCTAAAGTAGCCAT | 53 ℃ |  |
| JS17-3-F | CACTTGCTCTGAATGTGAACGA | 55 ℃ | 1472 bp |
| JS17-3-R | ACTGCCATTCTTGTTTCCTGA | 53 ℃ |  |
| JS17-4-F | TGATAAGAATCCTGTGTAAGCC | 53 ℃ | 1415 bp |
| JS17-4-R | GAATTGTAATAGGACTCAATGCAG | 54 ℃ |  |
| JS17-5-F | CTAGTAAAGGCAAATGAAAATGCTG | 54 ℃ | 1478 bp |
| JS17-5-R | GGAACAATGAAGTTTACAAACGGAA | 54 ℃ |  |
| JS17-6-F | TTATCATAATTCAGCATTCCACCAC | 54 ℃ | 1350 bp |
| JS17-6-R | CAACCTAAATAATATACCCCGCTTC | 56 ℃ |  |
| JS17-7-F | TTGTATGATGTACTGTTTCGTC | 52 ℃ | 1255 bp |
| JS17-7-R | TAAGTTAGACTCTTCATGTGCTA | 52 ℃ |  |
| JS17-8-F | GATATTACTCCCTGAAGTCCA | 53 ℃ | 1424 bp |
| JS17-8-R | TGATTAGGCTTCTTCTAAACACA | 52 ℃ |  |
| JS17-9-F | TAATGCTTCAAAGGGTCTCACGGAA | 57 ℃ | 1444 bp |
| JS17-9-R | ATCTCCAGGAATTGACACTTGAGG | 57 ℃ |  |
| JS17-10-F | TTCTATCAGGGGAGGATTCTAACGCAA | 59 ℃ | 1338 bp |
| JS17-10-R | TGTGCCATCATCCAACCGAT | 55 ℃ |  |
| JS17-11-F | GATAATCCTCCTATCCGTGTACCG | 59 ℃ | 1434 bp |
| JS17-11-R | GACTGCTACTTGACCCGACT | 57 ℃ |  |
| JS17-12-F | TACCAAAGATTAAGGGGTTCTCTCC | 57 ℃ | 1330 bp |
| JS17-12-R | TTGTTCAAATTCTATGAAAGTTGCG | 53 ℃ |  |
| JS17-13-F | TATTTAATTTGTATCCTTGCCAAC | 51 ℃ | 820 bp |
| JS17-13-R | GATATATTTAGATTTCCTCGCCATC | 54 ℃ |  |

[Supplementary](https://www.nature.com/articles/s41586-020-3035-9#MOESM1) Table 3. Primers used for PIV5-JS17 strain 5'/3' RACE

| **Names** | **Sequences (5' -3')** | **Annealing temperature (℃)** | **Length of PCR products** |
| --- | --- | --- | --- |
| 5' GSP1 | GCAGCAGGTTGGTCTGGCG | 63 | 720 bp |
| 5' NGSP1 | AAGCGATGGGAATCCCTTGC | 60 | 360 bp |
| 3' NGSP2 | GAAGCAGGACTTGGAATTCGGCATA | 62 | 366 bp |
| 3' GSP2 | ATCGTAGCAGAAGCAATTGATAAGG | 58 | 730 bp |

[Supplementary](https://www.nature.com/articles/s41586-020-3035-9#MOESM1) Table 4. Primers used for relative RT-qPCR

| **Names** | **Sequences (5'-3')** |
| --- | --- |
| eGFP-F | CAGGAGCGCACCATCTTCTT |
| eGFP-R | CGATGCCCTTCAGCTCGAT |
| rPIV5-M-F | TCGCCACTTATGAAGGTCCA |
| rPIV5-M-F | ACTGCCATTCTTGTTTCCTGA |
| GAPDH-F | TCGGAGTCAACGGATTTGGT |
| GAPDH-R | ACATGTAAACCATGTAGTTGAGGT |

[Supplementary](https://www.nature.com/articles/s41586-020-3035-9#MOESM1) Table 5. Primers used for quantitative RT-qPCR

| **Names** | **Sequences (5'-3')** |
| --- | --- |
| aPIV5-M-F | TGAAGTGTCGATTGCAGATATGTG |
| aPIV5-M-R | CCACCCTTTCCAAAAAACAACT |
| Probe | FAM-CATAATTCATGCCAGAGGTCACATTCCCA-BHQ1 |
